# Oxidative stress-driven cellular heterogeneity in Knee Osteoarthritis: scRNA-seq pinpoints *SPP1* and *CYBA* for diagnostic development

**DOI:** 10.1371/journal.pone.0360537

**Published:** 2026-10-27

**Authors:** Yijiang Liu, Yiman Chi, Jiefeng Yin, Jun Yao

**Affiliations:** 1 Bone and Joint Surgery, The First Affiliated Hospital of Guangxi Medical University, Nanning, Guangxi, China; 2 Department of General Practice, Yulin City First People’s Hospital, Yulin, Guangxi, China; 3 Bone and Joint Surgery, The First Affiliated Hospital of Guangxi Medical University, Nanning, Guangxi, China; University of Vermont College of Medicine, UNITED STATES OF AMERICA

## Abstract

Knee osteoarthritis (KOA) is a progressive degenerative joint disease characterized by cartilage degeneration, synovial inflammation, and subchondral bone remodeling. Oxidative stress plays a critical role in KOA pathogenesis. This study aimed to identify oxidative stress–related hub genes by integrating single-cell RNA sequencing (scRNA-seq) with machine learning to uncover potential diagnostic and prognostic biomarkers for KOA. ScRNA-seq data from KOA cartilage tissues were obtained from the GEO database to characterize cellular heterogeneity and identify disease-associated chondrocyte subsets. CellChat was used to analyze intercellular communication, and Monocle2 was applied to reconstruct cell differentiation trajectories. Oxidative stress activity at the single-cell level was evaluated using AUCell, while high-dimensional weighted gene co-expression network analysis (hdWGCNA) was performed to identify oxidative stress–related gene modules. Candidate genes were screened using Venn analysis and further refined by LASSO regression, SVM-RFE, and XGBoost combined with SHAP analysis. Diagnostic performance was assessed, and biological relevance was explored through immune infiltration and pathway analyses. Experimental validation was conducted using qRT-PCR and western blot assays in H_2_O_2_-treated chondrocytes.

InfC and ProFC were detected specifically in OA cartilage and exhibited high oxidative stress activity. InfC was enriched in inflammatory and immune-related pathways, whereas ProFC was associated with cell cycle regulation and extracellular matrix remodeling. Integrated hdWGCNA and machine-learning analyses consistently identified CYBA and SPP*1* as candidate genes. The two-gene model achieved bootstrap-corrected AUCs of 0.937 and 0.825 in the training and validation cohorts, respectively. qRT-PCR and western blot analyses further showed increased *CYBA* and *SPP1* expression in H_2_O_2_-treated chondrocytes.

Conclusion

InfC and ProFC may represent OA-associated chondrocyte states characterized by enhanced oxidative stress, while CYBA and SPP*1* may serve as candidate diagnostic biomarkers. Given the small cohort sizes and preliminary in vitro validation, these findings require confirmation in larger independent cohorts and functional studies.

## Introduction

Knee osteoarthritis (KOA) is one of the most prevalent degenerative joint disorders in adults. With an aging population and lifestyle changes, its incidence has been steadily increasing, posing a significant global public health burden [1]. KOA is a complex condition influenced by multiple factors, including genetic and epigenetic susceptibility, aging, diet and obesity, and chronic mechanical stress on the joints [2]. The pathology involves progressive cartilage loss, continuous synovial inflammation, changes in subchondral bone, and ongoing injury to adjacent tissues. Because joint structural damage is largely irreversible, there is currently no definitive treatment capable of halting or reversing disease progression. Existing therapeutic strategies primarily focus on delaying disease advancement, alleviating symptoms, and improving joint function. In advanced stages, patients often require total knee arthroplasty to restore quality of life [3]. Consequently, achieving sensitive and reliable early detection and intervention remains a major challenge. The identification and validation of molecular biomarkers for early diagnosis could provide novel avenues for personalized management and precision therapy in KOA.

Oxidative stress is recognized as a key contributor to cartilage degradation and inflammation in the progression of KOA [4]. It arises from disrupted redox homeostasis, caused by excessive production and insufficient clearance of reactive oxygen species (ROS), which in turn induce oxidative damage to cellular components (CCs) [5]. ROS are primarily generated through physiological systems such as mitochondrial metabolism, NADPH oxidases, and xanthine oxidase [6]. Under normal conditions, physiological ROS levels participate in intracellular signal transduction; however, when redox balance is disturbed, excessive ROS can trigger DNA damage, protein misfolding, and structural injury to cellular membranes and mitochondria [6,7]. Mitochondrial dysfunction further impairs respiratory chain activity, amplifying ROS production and creating a vicious cycle of oxidative dysregulation. These alterations promote chondrocyte apoptosis and reduce collagen synthesis. Additionally, oxidative stress can activate the classical NF-κB signaling pathway, leading to sustained production of pro-inflammatory cytokines, such as interleukin-1β (IL-1β) and tumor necrosis factor (TNF)-α, which exacerbate synovial inflammation and cartilage damage [8]. Moreover, oxidative stress may influence OA pathogenesis by modulating immune and inflammatory responses as well as apoptotic pathways through mitochondrial mechanisms [9].

Single-cell RNA sequencing (scRNA-seq) has emerged as a powerful tool for investigating the pathogenesis of KOA. Compared with traditional bulk RNA sequencing, scRNA-seq provides higher resolution at the transcriptomic level, enabling analysis of gene expression patterns at the single-cell scale [10]. scRNA-seq studies have demonstrated that cartilage tissue comprises multiple functionally specialized chondrocyte subtypes rather than a homogeneous population [11–14], challenging the traditional view of chondrocyte uniformity. Despite these advances, investigations into the impact of oxidative stress on gene expression profiles and regulatory pathways in OA at the single-cell level remain limited. In this study, we leveraged the high resolution of scRNA-seq to identify chondrocyte-specific markers and systematically analyze the composition of chondrocyte subpopulations. By integrating robust bioinformatics approaches, we further explored the functional effects of oxidative stress across different chondrocyte subsets. Finally, using advanced machine learning algorithms, we identified two core oxidative stress-related genes and constructed a diagnostic model with strong generalizability for OA.

We analyzed the associations between core genes, immune cell infiltration, and the local immune microenvironment, and further assessed their links with TNF family members and chemokine signaling. In vitro experiments supported the reliability of our bioinformatics results. Overall, integrating single-cell transcriptomics with machine learning not only deepens understanding of oxidative stress in osteoarticular diseases but also highlights oxidative stress–related genes as potential biomarkers. These findings may support early OA diagnosis and provide new directions for personalized treatment strategies.

## Materials and methods

### Data collection

We used the single-cell dataset from the GEO database (GSE169454, https://www.ncbi.nlm.nih.gov/geo/), which includes transcriptomic profiles of three healthy and four OA cartilage samples generated using the 10X Genomics platform (GPL16791, Illumina HiSeq 2500). The bulk RNA-seq dataset GSE57218 served as the training cohort (7 control knee cartilage and 33 OA samples), and GSE114007 as the validation cohort (18 control and 20 OA knee cartilage samples). The data used in this study were obtained from publicly available databases and were fully de-identified. The authors did not have access to information that could identify individual participants. Available clinical characteristics of the training and validation cohorts extracted from the original GEO records and associated publications are summarized in S1 Table.

### Processing of single-cell RNA-seq data

Single-cell data analysis was performed using Seurat (version 4.2.2). After data import, quality control (QC) measures were applied to remove cells with a mitochondrial gene proportion >5%, ribosomal gene proportion >40%, hemoglobin gene proportion (>1%, percent.HB), or nFeature_RNA values <500 or >6000. Data normalization and scaling were conducted using the SCTransform function. Dimensionality reduction and clustering were performed using the RunUMAP function based on the top 17 principal components. Cell type annotation was conducted using SingleR, based on published literature. Marker genes for each cell type were identified with the FindAllMarkers function. Potential doublets, which accounted for 7% of the cells, were detected using the scDblFinder function and subsequently removed.

### Cell–cell communication analysis

Cell–cell communication between OA and normal samples was analyzed using CellChat (v1.4.0). A curated ligand–receptor database guided the analysis. Expression data were preprocessed as required, and pathway-level communication probabilities were calculated based on ligand–receptor pairs. Communication was quantified by both the number and strength of interactions.

### Pseudotime trajectory analysis

We applied Monocle2 for pseudotime analysis to map the developmental trajectories of chondrocytes in OA. Expression data, cell annotations, and reduced-dimensional coordinates were transferred from Seurat to build a CellDataSet. Highly variable genes served as ordering features, and DDRTree was used for dimensionality reduction. Cells were arranged along pseudotime with the orderCells function, setting a specific root state. Visualization included trajectory plots, cell type–annotated trajectories, and pseudotime density plots to display the dynamic distribution of chondrocyte subtypes.

### AUCell scoring

A total of 1,491 oxidative stress–related genes (Relevance Score > 8) were obtained from GeneCards to generate the oxigene set (S1 Dataset). AUCell was then used to evaluate single-cell activity and calculate enrichment scores for each cell. The distributions were shown with violin and box plots, and cells were divided into high- and low-score groups based on the median. Differentially expressed genes between groups were identified with Seurat and ranked by log2FC. Gene set enrichment analysis (GSEA) was performed using clusterProfiler with MSigDB, and significant pathways were visualized via gseaplot2.

### hdWGCNA analysis

The hdWGCNA package was used to perform weighted gene co-expression network analysis (WGCNA), covering network construction, module detection, and functional annotation. A normalized metacell matrix was generated with MetacellsByGroups, and the optimal soft-thresholding power was determined by TestSoftPowers based on scale-free topology criteria. Networks were then constructed with ConstructNetwork, modules were automatically detected (excluding the “grey” module), and gene module membership (kME) was calculated for downstream feature selection and annotation.

### Single-cell GO and KEGG enrichment analysis

Marker genes of each chondrocyte subpopulation were identified with Seurat (*p* < 0.01, |log_2_FC| > 1). GO and KEGG enrichment analyses were performed using clusterProfiler, and pathways with adjusted *p* < 0.05 were considered significant for downstream interpretation.

### Machine learning–based selection of oxidative stress–related hub genes

Three machine learning algorithms were applied for feature selection: LASSO regression, SVM-RFE, and XGBoost with SHAP. LASSO was performed with 10-fold cross-validation, and genes with non-zero coefficients were retained. SVM-RFE with an RBF kernel was used to iteratively eliminate features, selecting the subset with the highest accuracy. XGBoost models were trained, and SHAP values quantified each gene’s contribution. Genes identified by all three methods were defined as hub genes and displayed using a Venn diagram.

### Construction and validation of the diagnostic model

Candidate genes were modeled with the rms package to build a logistic regression diagnostic model. Predicted probabilities were calculated using the predict function, and model performance was assessed with ROC curves (pROC) and AUC values. AUCs with 95% confidence intervals were estimated for the training and validation cohorts using 1,000 bootstrap resamples. Calibration plots assessed agreement between predicted and observed probabilities. The optimal probability cutoff was determined in the training cohort using the Youden index and then applied to both cohorts. Sensitivity, specificity, positive predictive value (PPV), and negative predictive value (NPV), with 95% confidence intervals calculated using the Clopper–Pearson exact method, were reported at this cutoff.A nomogram was generated to visualize variable contributions, and Decision curve analysis (DCA, rmda package) was used to assess clinical value.

### Immune-related analysis

The IOBR package(version 0.99.0)was applied to estimate immune cell composition using MCP-counter, CIBERSORT (version 0.1.0), and ESTIMATE, with results integrated into an immune score matrix [15]. For CIBERSORT, the LM22 signature matrix comprising 547 genes and 22 immune cell types was applied with 1,000 permutations, and samples with *P* < 0.05 were retained. Differences in immune infiltration between OA and controls were compared using t-tests. CIBERSORT calculated the proportions of 22 immune cell types, and correlations between hub genes and immune cells were examined with bubble plots. Spearman correlation was used to assess associations with TNF/TNFR family members and chemokines, while butterfly plots (tidyquant, ggplot2) displayed positive and negative correlation patterns.

### Drug prediction

Potential drug targets for the hub genes *CYBA* and *SPP1* were explored using the Drug–Gene Interaction Database (DGIdb, https://dgidb.org), which was searched for relevant drug–gene associations. Interaction scores were extracted from the results, and the Python seaborn visualization package was used to generate a heatmap of gene–drug interaction scores, providing an intuitive representation of the interaction strength between hub genes and various drugs.

### Cell culture and treatment

Healthy 3–7-day-old Sprague–Dawley rats were obtained from Guangxi Medical University. Rats were anesthetized with isoflurane and euthanized by intraperitoneal injection of sodium pentobarbital. Under sterile conditions, knee cartilage was collected, minced, and digested with trypsin (30 min) followed by type II collagenase (1 mg/mL, 6 h) in high-glucose DMEM. The digested suspension was centrifuged, and the pellet was resuspended in DMEM supplemented with 10% FBS and penicillin/streptomycin. Cells were cultured at 37 °C with 5% CO_2_, and third-passage (P3) chondrocytes were used. For in vitro OA modeling, chondrocytes were stimulated with H_2_O_2_ (40 μmol/L) for 12 h. Expression of *MMP13* and Col2a1 was measured to validate OA induction.

### RNA extraction and quantitative real-time PCR (qRT-PCR)

Total RNA was extracted from chondrocytes using the RNeasy Plus kit (Beyotime, China) and reverse-transcribed into cDNA with PrimeScript RT Reagent Kit (Takara, China). qRT-PCR was conducted to detect *MMP13*, Col2a1, *CYBA*, and *SPP1* expression, with primers designed from GenBank (Table 1). Each reaction (20 μL) contained SYBR Premix Ex Taq, primers, sterile water, and cDNA template. *Gapdh* was used as the reference gene, and relative expression was calculated using the 2^ − ΔΔCt method, with the mean ΔCt of the control group as the calibrator. Data are presented as mean ± SD, and between-group differences in ΔCt values were assessed using paired *t*-test.

**Table 1:** Primer sequences for qRT-PCR analysis.

Gene symbol	Species	Forward primer (5’ → 3’)	Reverse primer (5’ → 3’)
*Col2a1*	Rat	GGGAATGTCCTCTGCGATGAC	GAAGGGGATCTCGGGGTTG
*Mmp13*	Rat	CTTCTTCTTGTTGAGCTGGACTC	CTGTGGAGGTCACTGTAGACT
*Spp1*	Rat	TGAGTTTGGCAGCTCAGAGG	TCGTCGTCGTCATCATCGTC
*Cyba*	Rat	GGACAGAAGTACCTGACCGC	GTAGGTGGCTGCTTGATGGT

### Western blot

Total proteins were isolated using RIPA buffer (Beyotime, China) supplemented with phosphatase and protease inhibitors (CWBIO, MCE) and PMSF (BOSTER, China). Protein concentration was determined with a BCA kit (Beyotime, China). Equal amounts of protein were denatured at 100 °C for 10 min, separated on 7.5% SDS–PAGE gels (ACE, China), and transferred onto nitrocellulose membranes. After blocking with 5% skim milk for 2 h, membranes were incubated overnight at 4 °C with primary antibodies against *SPP1* (Proteintech, Cat. No. 83341–1-RR; 1:5,000), *MMP13* (Proteintech, Cat. No. 18165–1-AP; 1:3,000), β-actin (Proteintech, Cat. No. 20536–1-AP; 1:5,000), *COL2A1* (Abcam, Cat. No. ab307674; 1:1,000), α-tubulin (Proteintech, Cat. No. 80762–1-RR; 1:8,000), and *CYBA* (Thermo Fisher Scientific, Cat. No. PA5–99812; 1:2,000). After three washes with PBST, the membranes were incubated with an HRP-conjugated secondary antibody (Proteintech, Cat. No. RGAR001; 1:10,000) at room temperature for 1 h. Protein bands were visualized using an enhanced chemiluminescence reagent (ECL; Beyotime, China) and imaged using an Odyssey imaging system, and quantified using ImageJ.

### Statistical analysis

Statistical analyses were performed using GraphPad Prism, SPSS (v24.0), and RStudio (v2024.12.1). Pearson correlation assessed associations between modules and traits, with significance defined as |r| > 0.8 and *p* < 0.05. Data are shown as mean ± SD. For normally distributed data with equal variance, independent-sample *t*-tes*t*s were used; otherwise, the Wilcoxon rank-sum test was applied. A *p*-value ≤ 0.05 was considered statistically significant.

### Ethics statement

This study was conducted using publicly available human transcriptomic data from the Gene Expression Omnibus (GEO) database. No new human participants were recruited, and no interventions or experiments were performed on human subjects. All data were fully de-identified prior to analysis, and no individual-level identifiable information was accessed. In accordance with Article 32 (Items 1–2) of the *Ethical Review Measures for Life Science and Medical Research Involving Humans* (China, 2023), this study is exempt from Institutional Review Board (IRB) review. All animal experiments were approved by the Ethics Committee of the First Affiliated Hospital of Guangxi Medical University (Approval No. 2025-E0659). All animal experiments were conducted after ethical approval had been obtained. Written informed consent was obtained for the publication of any potentially identifiable data or images.

## Results

### Cellular composition and characteristics of OA chondrocytes

Following integration and quality control of the single-cell data, a total of 49,848 chondrocytes were obtained, including 42,324 from OA samples and 7,524 from normal samples. After batch correction, dimensionality reduction, and clustering, 17 cell clusters were identified. Based on SingleR annotation and previously published literature [13,14], these clusters were classified into 11 chondrocyte subtypes (Fig 1A). The subtypes and their representative marker genes were as follows (Fig 1B): Homeostatic chondrocytes (HomC): *HSPA1A*, HSPA1B, HSPA6, DDIT3, *JUN* regulatory chondrocytes (RegC): *CHI3L1*, CHI3L2 proliferative chondrocytes (ProC): C11orf96, BMP2, HMGA1 prehypertrophic chondrocytes (preHTC): *PRG4*, ABI3 BP, CRTAC1 inflammatory chondrocytes (InfC): CXCL8, CD74, GPR183 HTC: *COL10A1*, *SPP1*, IBSP effector chondrocytes (EC): FRZB, CYTL1, CHRDL2 reparative chondrocytes (RepC): CILP, CILP2, OGN fibrocartilage chondrocytes (FC): MMP2, *COL1A2*, *COL1A1* Proliferative FC (ProFC): *STMN1*, KIAA0101, H2AFZ PreFC (preFC): COL27A1, PLCG2, WWP2.

**Fig 1:**
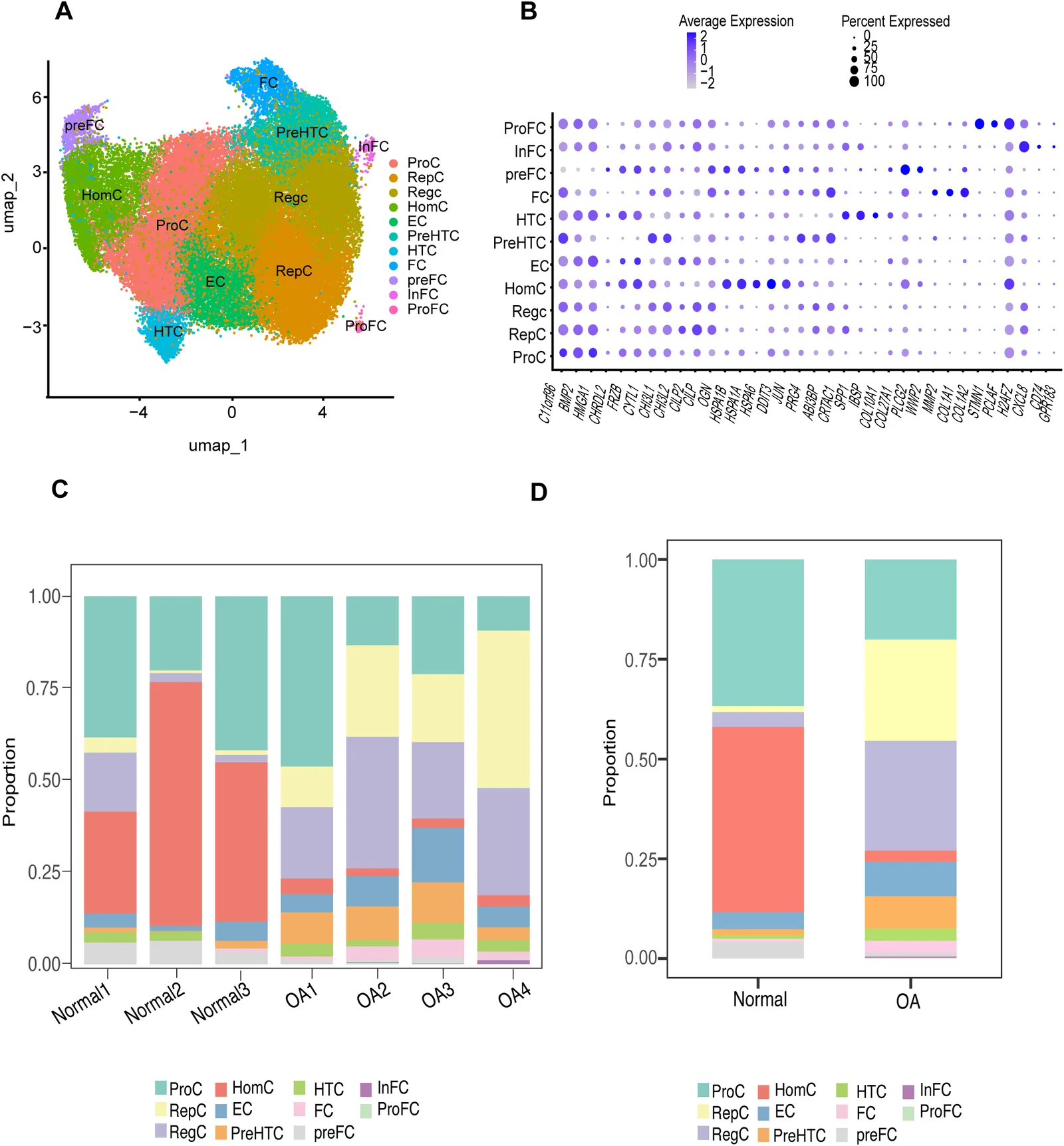
Identification of chondrocyte subtypes and compositional changes in OA and normal samples. (A) UMAP projection of combined single-cell transcriptomes from normal and OA chondrocytes revealed 11 distinct chondrocyte subtypes. (B) Bubble plot displaying marker genes of each subtype, with dot size indicating the fraction of positive cells and color denoting mean expression levels. (C) Distribution of chondrocyte subtypes across different samples. (D) Proportion of chondrocyte subtypes in normal and OA groups.

To investigate OA-related changes in chondrocyte composition, we compared the distribution of each subtype across individual cartilage samples (Fig 1C) and between normal and OA groups (Fig 1D). Compared with normal cartilage, OA samples exhibited marked increases in RepC, RegC, EC, preHTC, HTC, and FC, alongside significant decreases in HomC, ProC, and preFC. Notably, InfC and ProFC were detected exclusively in OA samples, with cell counts of 164(0.38%) and 86 (0.2%), respectively. Given the distinct heterogeneity of InfC and ProFC, we performed gene ontology (GO) and Kyoto encyclopedia of genes and genomes (KEGG) pathway enrichment analyses on the characteristic genes of each subset. At the GO level, InfC genes were primarily enriched in biological processes (BPs) associated with immune cell migration, chemotaxis, endocytosis/granule organization, and chemokine receptor activity, indicating a close involvement in immune regulation (Fig 2A). KEGG analysis revealed that InfC genes were significantly enriched in multiple immune- and inflammation-related signaling pathways, including NF-κB, chemokine, IL-17, and Toll-like receptor signaling, as well as pathways related to rheumatoid arthritis (RA) and viral infections, suggesting a central role for InfC in inflammatory and immune response mechanisms (Fig 2B). ProFC genes, by contrast, were mainly enriched at the GO level in processes related to chromosome segregation, mitosis, and spindle assembly, and in molecular functions (MFs) such as microtubule/chromosome binding, transcriptional regulation, and protease activity, implying a role in cell division, chromosome dynamics, and regulatory mechanisms (Fig 2C). At the KEGG level, ProFC genes were predominantly enriched in pathways governing cell cycle regulation, DNA replication, and DNA repair, indicating that ProFC may contribute to cell proliferation and the maintenance of genetic stability (Fig 2D).

**Fig 2:**
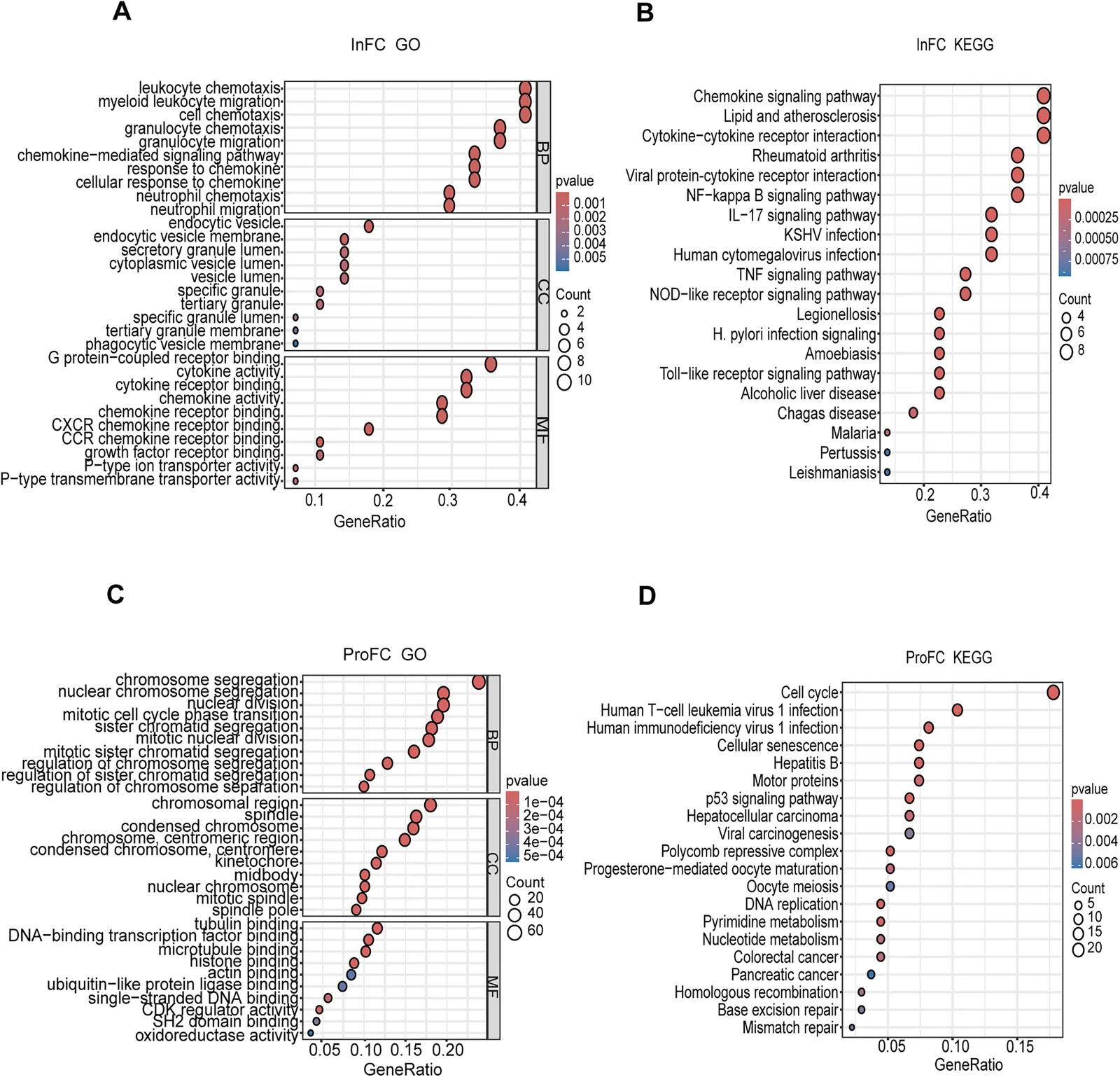
Functional enrichment of InfC and ProFC in OA and normal cartilage. (A) GO functional enrichment of InfC marker genes. (B) KEGG pathway enrichment for InfC marker genes. (C) GO functional enrichment of ProFC marker genes. (D) KEGG pathway enrichment of ProFC marker genes.

### Cell-Cell communication and pseudotime trajectory analysis

Cell-cell communication analysis (Figs 3A and 3D) demonstrated that, compared with the normal group, both the number and strength of intercellular interactions were markedly increased in OA chondrocytes. Communication activity was significantly enhanced in RepC and RegC, whereas interactions mediated by HomC were notably reduced. Of particular interest, ProFC emerged as the most active signaling hub in OA cartilage, receiving inputs from multiple cell populations while transmitting extensive regulatory signals outward. Analysis of incoming and outgoing signaling patterns (Figs 4A and Fig 4B) revealed that intercellular communication was predominantly mediated by the COLLAGEN and fibronectin 1 (*FN1*) signaling pathways. Heatmap analysis (Figs 4C and 4D) further indicated that InfC primarily functioned as a downstream receptor regulated by collagen- and fibronectin-associated signals, whereas ProFC acted as the major source of output for these pathways. ProFC not only influenced multiple chondrocyte subpopulations but also retained signal-receiving capabilities, highlighting its role as a central hub in cartilage extracellular matrix (ECM) remodeling and intercellular communication.

**Fig 3:**
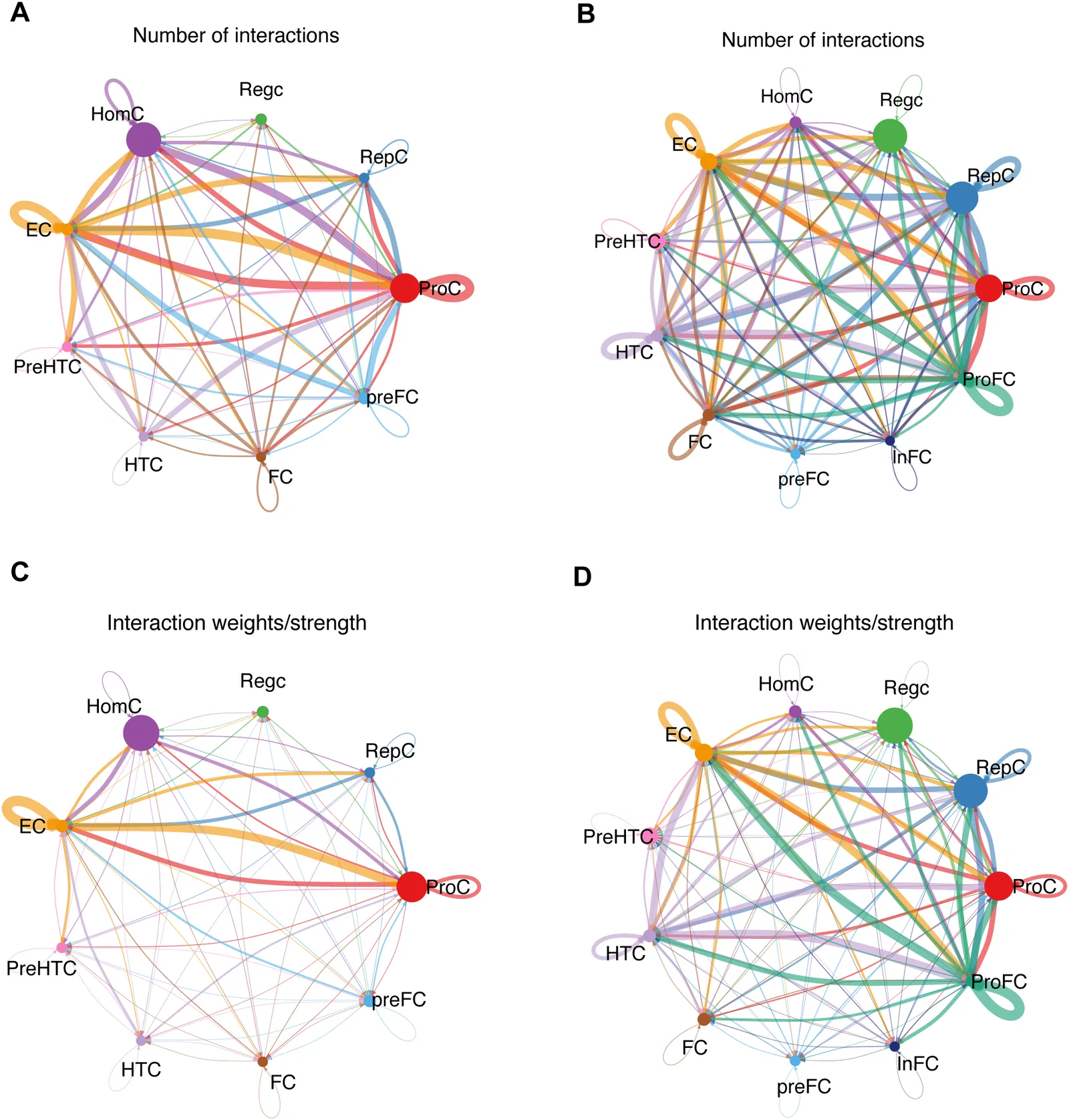
Cell–cell communication analysis. (A, B) Number and intensity of intercellular communication networks in normal cartilage. (C, D) Number and intensity of intercellular communication networks in OA cartilage.

**Fig 4:**
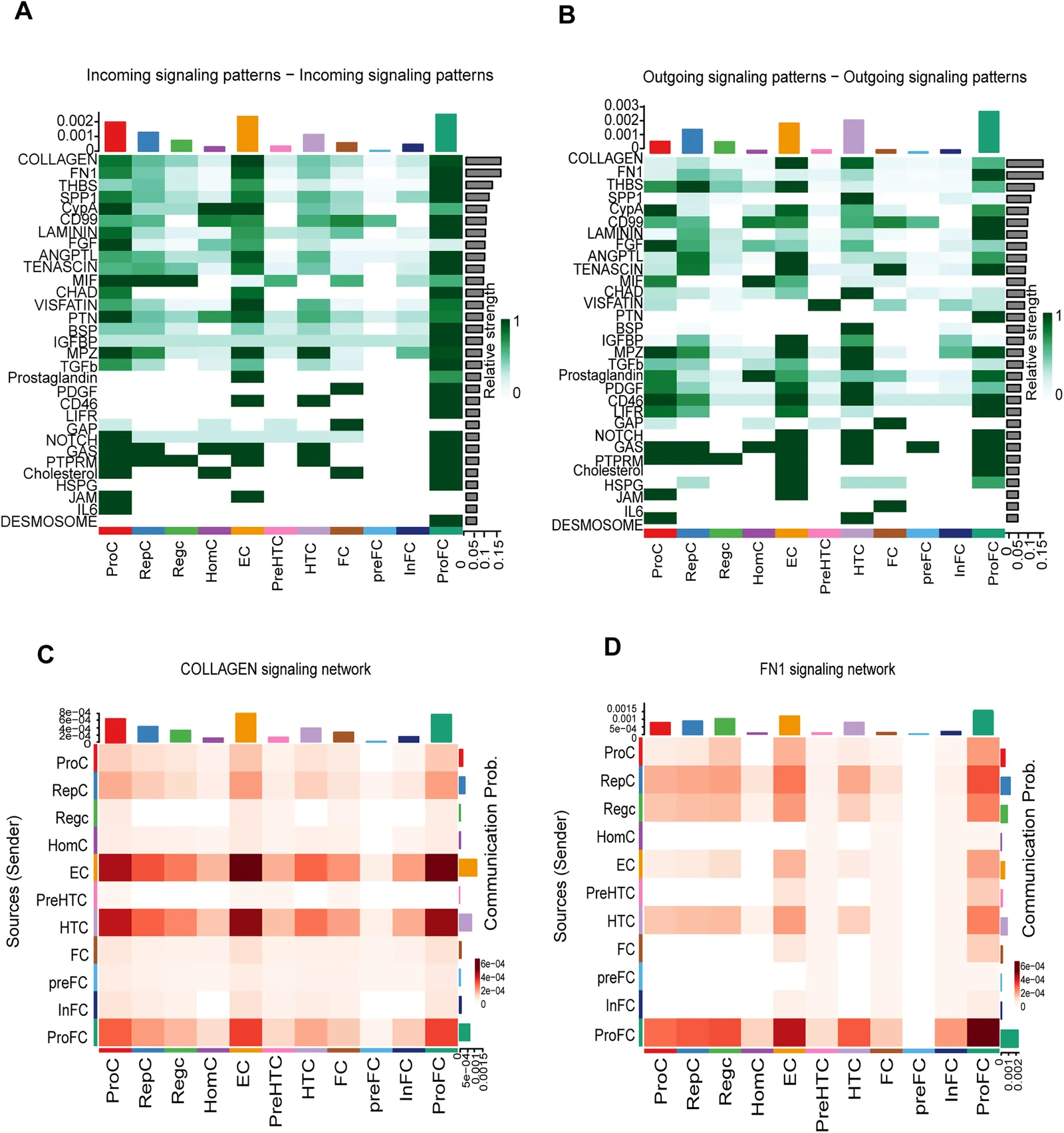
CellChat analysis of signaling patterns and extracellular matrix communication among OA chondrocytes. (A) Heatmap of incoming signaling patterns. (B) Heatmap of outgoing signaling patterns. (C) Heatmap of COLLAGEN pathway activity across various chondrocyte subsets. (D) Heatmap of *FN1* pathway activity across distinct chondrocyte subsets.

In the Monocle-constructed trajectory (Figs 5A and 5B), early subpopulations, including ProC and RepC, were primarily enriched at the initial stages of pseudotime, whereas FC, ProFC, and InfC were predominantly located at the terminal stage, indicating that they represent terminally differentiated subsets. This dynamic distribution pattern illustrates the continuous, stepwise differentiation of chondrocyte subtypes within the OA microenvironment.

**Fig 5:**
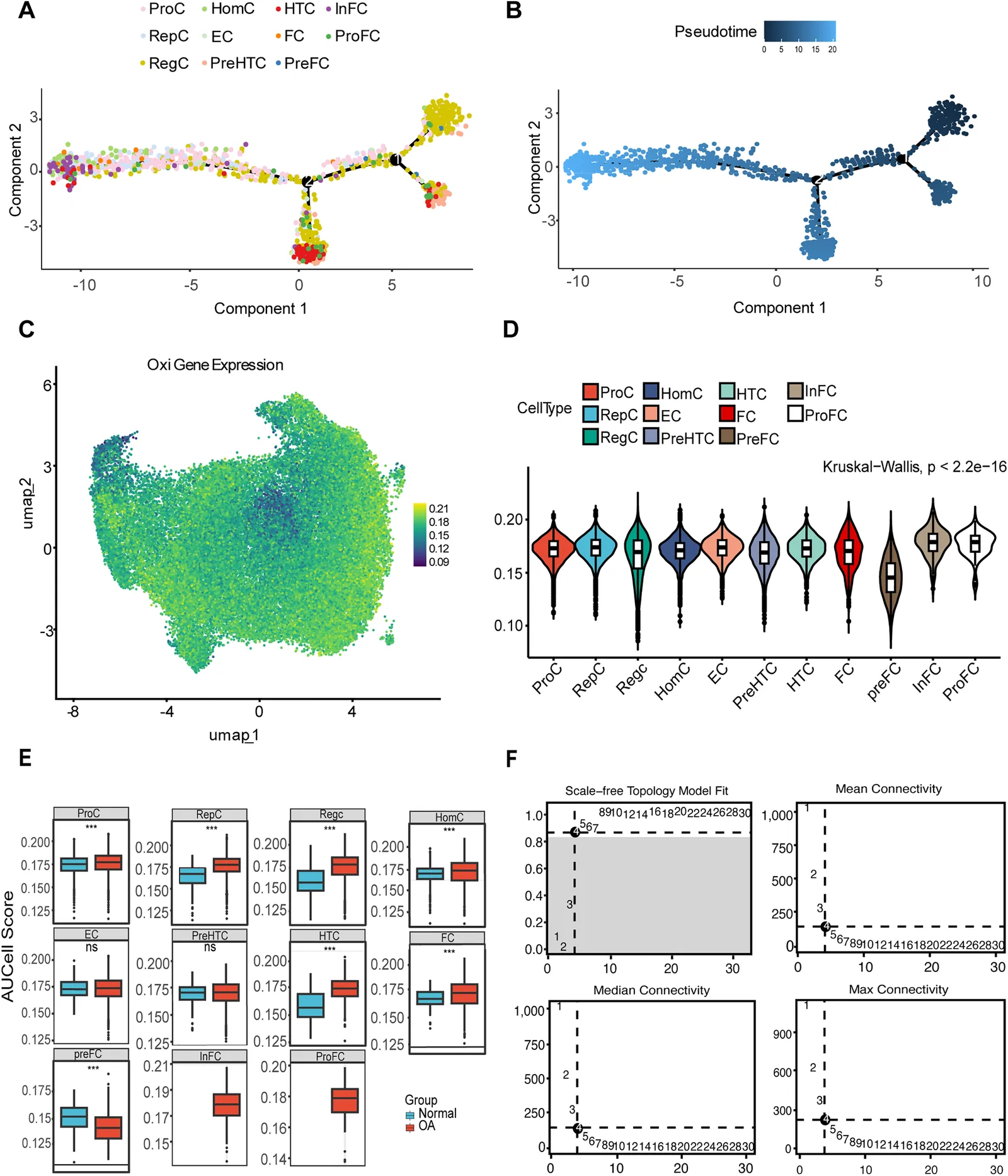
Pseudotime analysis, oxidative stress scoring analysis, and hdWGCNA module identification. (A) Pseudotime trajectory constructed using Monocle, illustrating the distribution of different cell subpopulations along the developmental trajectory. (B) Pseudotime plot constructed using Monocle, with color gradients from light to dark representing the progression along the trajectory. (C)Oxidative stress–related scoring of single cells using the AUCell algorithm, visualized in UMAP space. Colors range from purple to yellow, indicating low to high scores. D) Violin plots showing oxidative stress scores across different cell subtypes in all samples. (E) Boxplots comparing oxidative stress AUCell scores across different cell subtypes in normal versus OA samples. (F) hdWGCNA analysis of soft-thresholding power selection, identifying the optimal power parameter as 4.

### Oxidative stress signature analysis based on AUCell scoring and functional enrichment

To evaluate the impact of oxidative stress in OA, we applied the AUCell algorithm to calculate oxidative stress-related scores at the single-cell level, using a curated set of oxidative stress-associated gene markers. The spatial distribution of these scores was visualized using UMAP (Fig 5C). Among all chondrocyte subtypes (Fig 5D), InfC and ProFC exhibited the highest oxidative stress scores, indicating a highly active oxidative state, whereas preFC displayed the lowest score. Compared with the normal group, all chondrocyte subtypes in OA cartilage showed increased oxidative stress scores, except for preFC, which decreased, and EC and preHTC, which exhibited no statistically significant changes (Fig 5E).

### Identification of core genes via hdWGCNA and multiple machine learning algorithms

To identify key oxidative stress-related genes and their associated functional modules, we performed a co-expression network analysis using the hdWGCNA algorithm. The optimal soft-thresholding power was determined to be 4 (Fig 5F). Using this threshold, a weighted gene co-expression network was built, resulting in the identification of multiple gene modules, each represented by a distinct color, with gray denoting genes not assigned to any module. Four major modules were ultimately identified (Figs 6A and 6B): M1 (blue), M2 (yellow), M3 (dark red), and M4 (cyan). Correlation bubble plots revealed that InfC was most strongly associated with genes in the M2 module, whereas ProFC was highly correlated with genes in M3 (Fig 6C). Module eigengene UMAP visualization further confirmed that M2 and M3 modules were predominantly enriched in InfC and ProFC populations, respectively (Fig 6D). Considering the high oxidative stress activity of these two subtypes in OA cartilage, as indicated by AUCell scores, we extracted 42 genes from the M2 and M3 modules with module-cell correlations greater than 0.1 for downstream analyses. GO enrichment analysis of these 42 genes (Fig 6E) revealed significant enrichment in inflammation- and immune-related processes. At the BP level, these genes were mainly associated with chemotaxis, leukocyte migration, and granulocyte chemotaxis. CC analysis indicated enrichment in the vesicle lumen, endoplasmic reticulum lumen, and collagen-containing ECM, while MF analysis highlighted cytokine activity, chemokine binding, and receptor activity, KEGG pathway analysis (Fig 6F) further demonstrated enrichment in cytokine-cytokine receptor interaction, TNF signaling, IL-17 signaling, Toll-like receptor signaling, and RA, suggesting that their potential involvement in inflammatory and immune regulation in OA.

**Fig 6:**
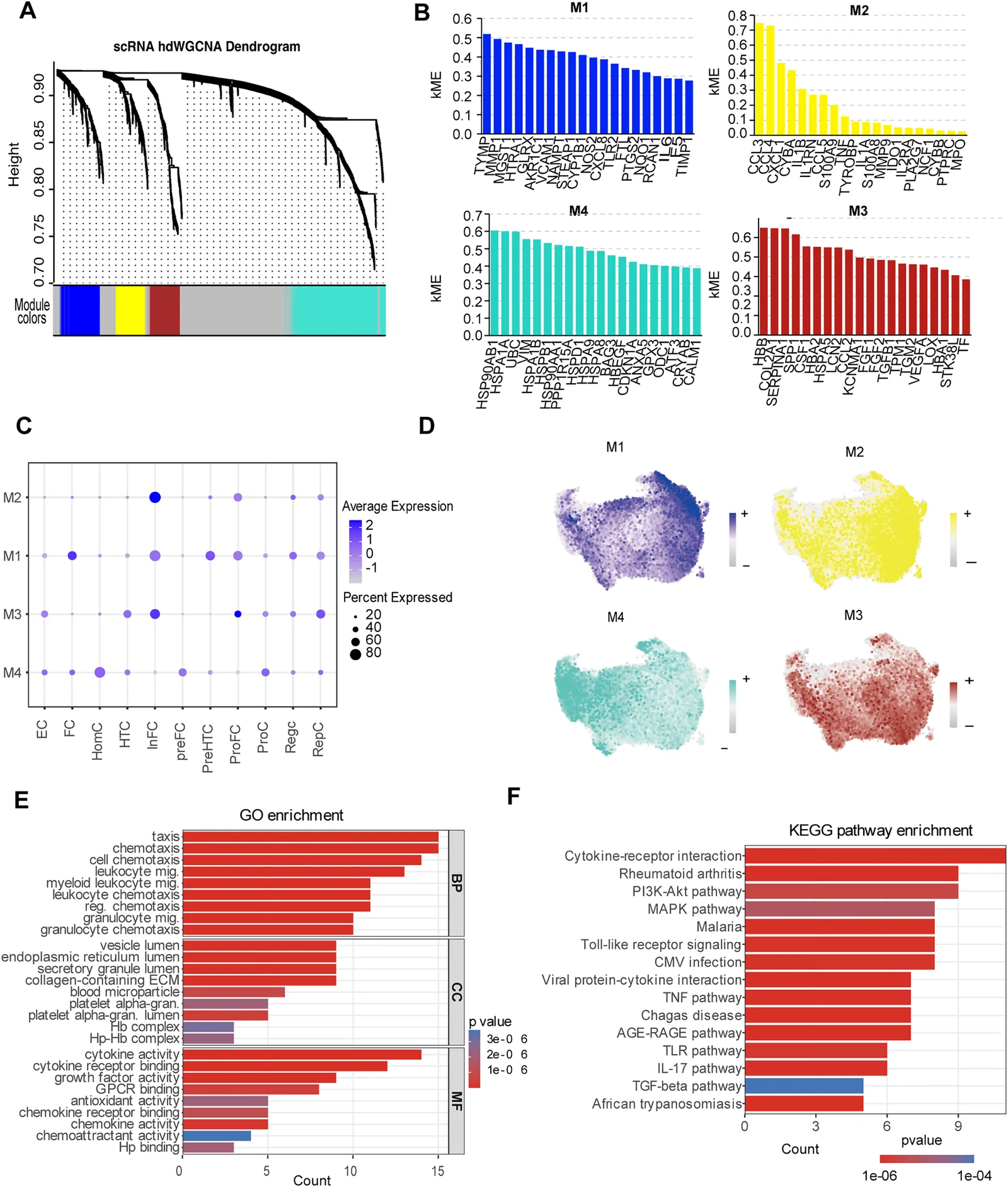
hdWGCNA analysis. (A) hdWGCNA module clustering dendrogram, where different colors represent distinct co-expression modules, and gray indicates genes not assigned to any module. (B) Bar plot illustrating the association between genes in individual modules and their representative eigengene, highlighting the key characteristic genes and the correlation strength in modules M1–M4. (C) Bubble plot of oxidative stress–related genes and cell subtypes. Bubble size = percentage of expressing cells; color = average expression level. (D) Distribution of module eigengenes (Module Eigengenes) in the UMAP space. (E) GO enrichment analysis of genes correlated with modules M2 and M3 (correlation > 0.1), separately displaying biological process (BP), cellular component (CC), and molecular function (MF) categories. (F) KEGG pathway enrichment was carried out for genes linked to modules M2 and M3.

Based on AUCell scoring, 658 differentially expressed genes (logFC > 0.25, *P* < 0.05; S2 Dataset) were identified, along with 42 hub genes derived from the M2 and M3 modules of hdWGCNA (S3 Dataset). Integration of these datasets using a Venn diagram revealed 16 overlapping oxidative stress-related genes associated with OA (Fig 7A). A functional interaction network centered on these 16 genes was constructed using GeneMANIA (Fig 7B), with the majority of connections arising from gene co-expression (59.19%) and physical interactions (31.87%). Functional annotation indicated that these genes were predominantly involved in BPs such as chemotaxis, hydrogen peroxide metabolism, ROS metabolism, cytokine receptor binding, and chemokine receptor interaction, highlighting strong functional connectivity among these candidate genes. To refine the core gene set, we employed three machine learning algorithms, LASSO regression (Fig 7C and 7D), SVM-RFE (Fig 7E and 7F), and XGBoost combined with SHAP analysis (Fig 8A). The complete outputs of LASSO, SVM-RFE, and XGBoost-SHAP analyses are provided in S4 Dataset. Integration of these results using a Venn diagram (Fig 8B) identified two consensus core genes: *CYBA* and *SPP1*. SHAP feature dependency analysis demonstrated that high expression levels of these genes strongly contributed to OA prediction, indicating their positive regulatory roles (Fig 8C and 8D). Boxplot analyses further confirmed that *CYBA* and *SPP1* were significantly upregulated in OA samples across both training and validation datasets (Fig 8E and 8F).

**Fig 7:**
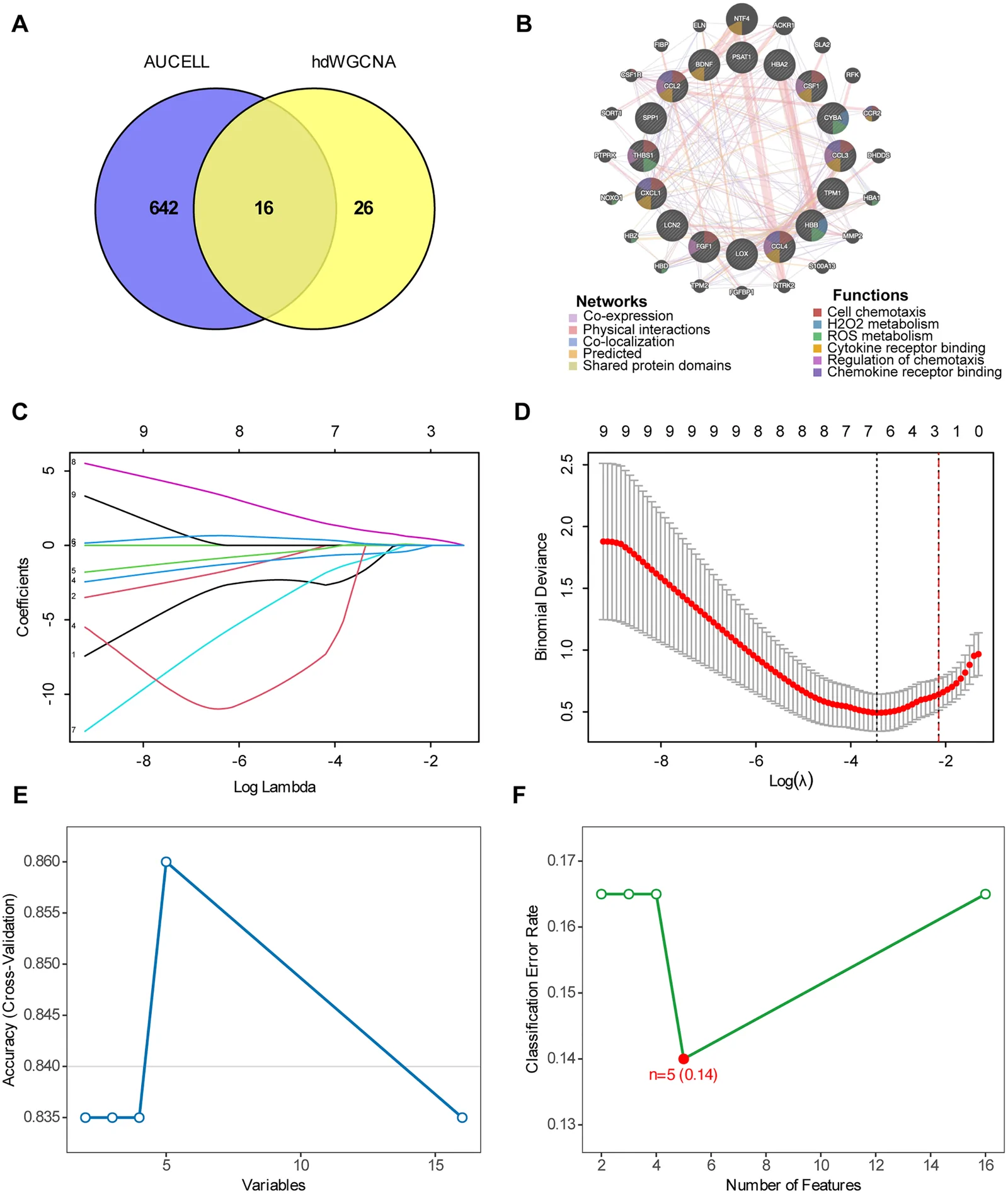
Machine learning–based screening of OA oxidative stress–related core genes. (A) A Venn diagram illustrated the intersection of AUCell-derived differentially expressed genes with hdWGCNA modules M2 and M3, revealing 16 shared genes related to OA oxidative stress. (B) Functional interaction network of oxidative stress core genes constructed using GeneMANIA, where different colors represent distinct functional categories and edge types indicate the corresponding interaction types. (C, D) Feature selection with the LASSO regression model: (C) coefficient profiles, and (D) cross-validated mean squared error curve used to identify the optimal penalty λ. (E, F) Support vector machine–recursive feature elimination (SVM-RFE) results, displaying (E) accuracy and (F) classification error rates across varying feature numbers, with the optimal feature set highlighted.

**Fig 8:**
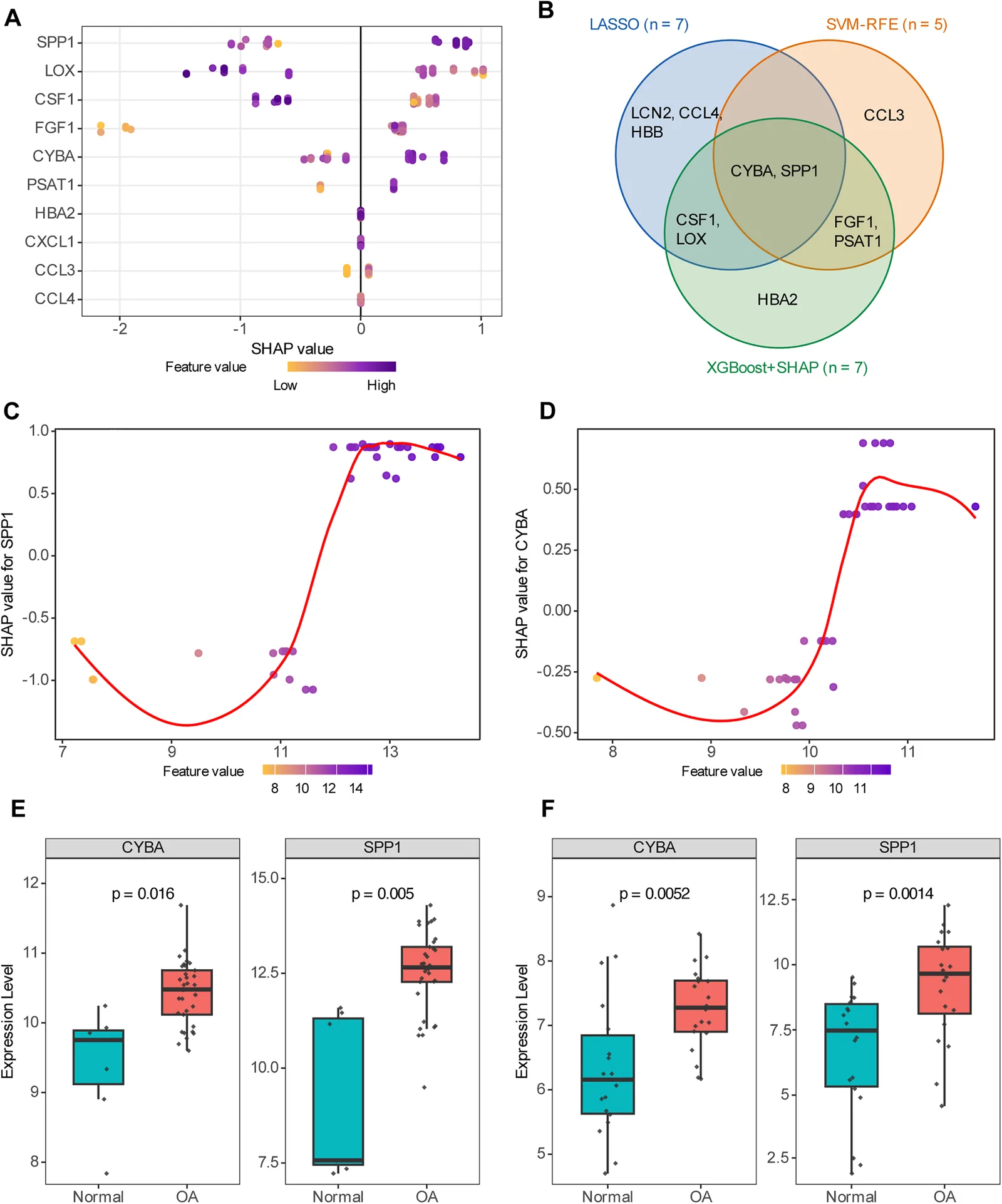
Machine learning-based identification and validation of *CYBA* and *SPP1* as oxidative stress-related diagnostic biomarkers. (A) Global SHAP beeswarm plot: the x-axis shows SHAP values (impact on predictions), the y-axis lists genes, dot colors indicate feature levels (yellow = low, purple = high), and horizontal spread reflects contribution magnitude and direction. (B) Venn diagram depicting overlapping genes detected through LASSO, SVM-RFE, and XGBoost + SHAP analyses.(C, D) SHAP dependence plots for *SPP1* and *CYBA*. The x-axis indicates gene expression, the y-axis shows SHAP values for OA risk contribution, the red line marks the LOESS trend, and dot colors denote feature value levels. (E, F) Boxplots illustrating *CYBA* and *SPP1* expression differences between OA and control groups across the training and validation datasets.

### Diagnostic performance of oxidative stress-related core genes

ROC analysis was used to evaluate the diagnostic performance of the CYBA–SPP*1* model. The bootstrap-corrected AUCs were 0.937 (95% CI: 0.681–0.971) in the training cohort and 0.825 (95% CI: 0.681–0.936) in the validation cohort (Fig 9A and 9B). Calibration was reasonable in the training cohort but showed some deviation in the validation cohort (S1 Fig). The optimal probability cutoff of 0.908 was determined in the training cohort using the Youden index and applied unchanged to the validation cohort. At this cutoff, sensitivity, specificity, PPV, and NPV were 84.8%, 100.0%, 100.0%, and 58.3%, respectively, in the training cohort, and 80.0%, 61.1%, 69.6%, and 73.3%, respectively, in the validation cohort; corresponding 95% CIs are shown in S1 Fig. These results indicate that the model retained moderate discriminatory ability in the independent cohort, although its performance was reduced. Nomograms were constructed to facilitate model interpretation, and DCA was performed in both cohorts (Figs 9C–9F). Because sparse events produced unstable estimates at very high threshold probabilities, clinical utility was interpreted primarily within the threshold range of 0.1–0.6. Within this range, the model showed potential net benefit; however, external validation, recalibration, and clinical utility assessment in larger independent cohorts and prospective clinical samples are required.

**Fig 9:**
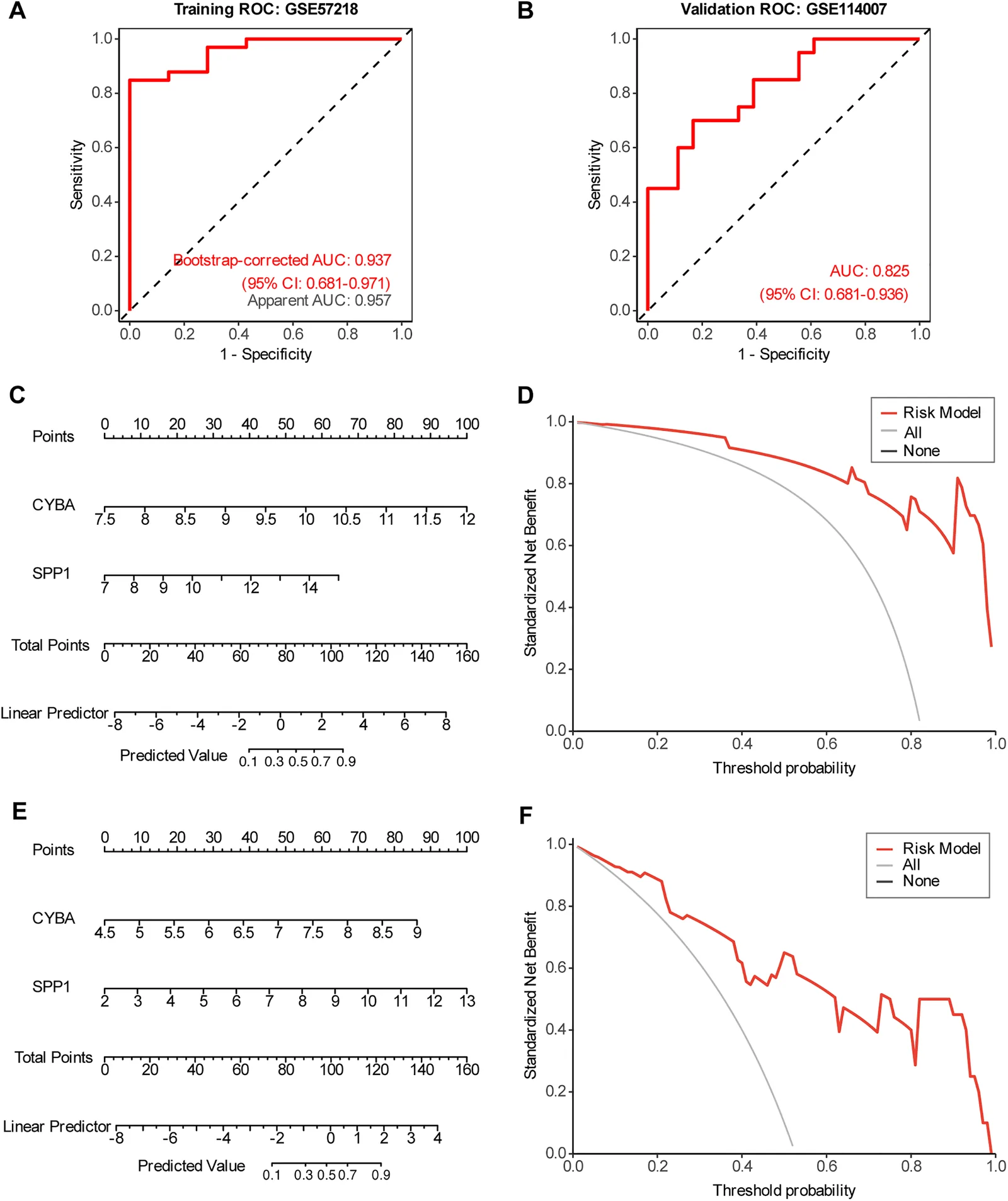
Nomogram, decision curve analysis (DCA) analysis of the *CYBA* and *SPP1* gene diagnostic model. (A, B) Receiver operating characteristic (ROC) curves of the combined diagnostic model using *CYBA* and *SPP1* in both training and validation cohorts, showing the model’s sensitivity and specificity. (C, D) Nomogram and decision curve analysis (DCA) of the diagnostic model in the training dataset. (E, F) Nomogram and DCA of the diagnostic model in the validation dataset.

### Immune-related functional analysis and drug target prediction of core genes

In the MCP-counter immune analysis (Fig 10A), endothelial cells and fibroblasts were significantly increased in the OA group, suggesting enhanced angiogenic and fibrotic signatures associated with immune infiltration. In the ESTIMATE analysis (Fig 10B), StromalScore, ImmuneScore, and ESTIMATEScore were all significantly elevated, whereas TumorPurity was reduced in OA samples, indicating alterations in the stromal and overall immune microenvironment. In the CIBERSORT analysis (Figs 10C and 10D), the OA group displayed elevated levels of resting NK cells and reduced levels of activated NK cells, while correlations among immune cell subsets remained generally moderate to low (Fig 11A). *CYBA* and *SPP1* expression levels were significantly positively correlated with ESTIMATEScore, StromalScore, endothelial cells, fibroblasts, and cytotoxic lymphocytes (Fig 11B). Collectively, these results indicate that abnormal expression of *CYBA* and *SPP1* could jointly drive immune microenvironment imbalance in OA, thereby facilitating persistent inflammation and disease progression.

**Fig 10:**
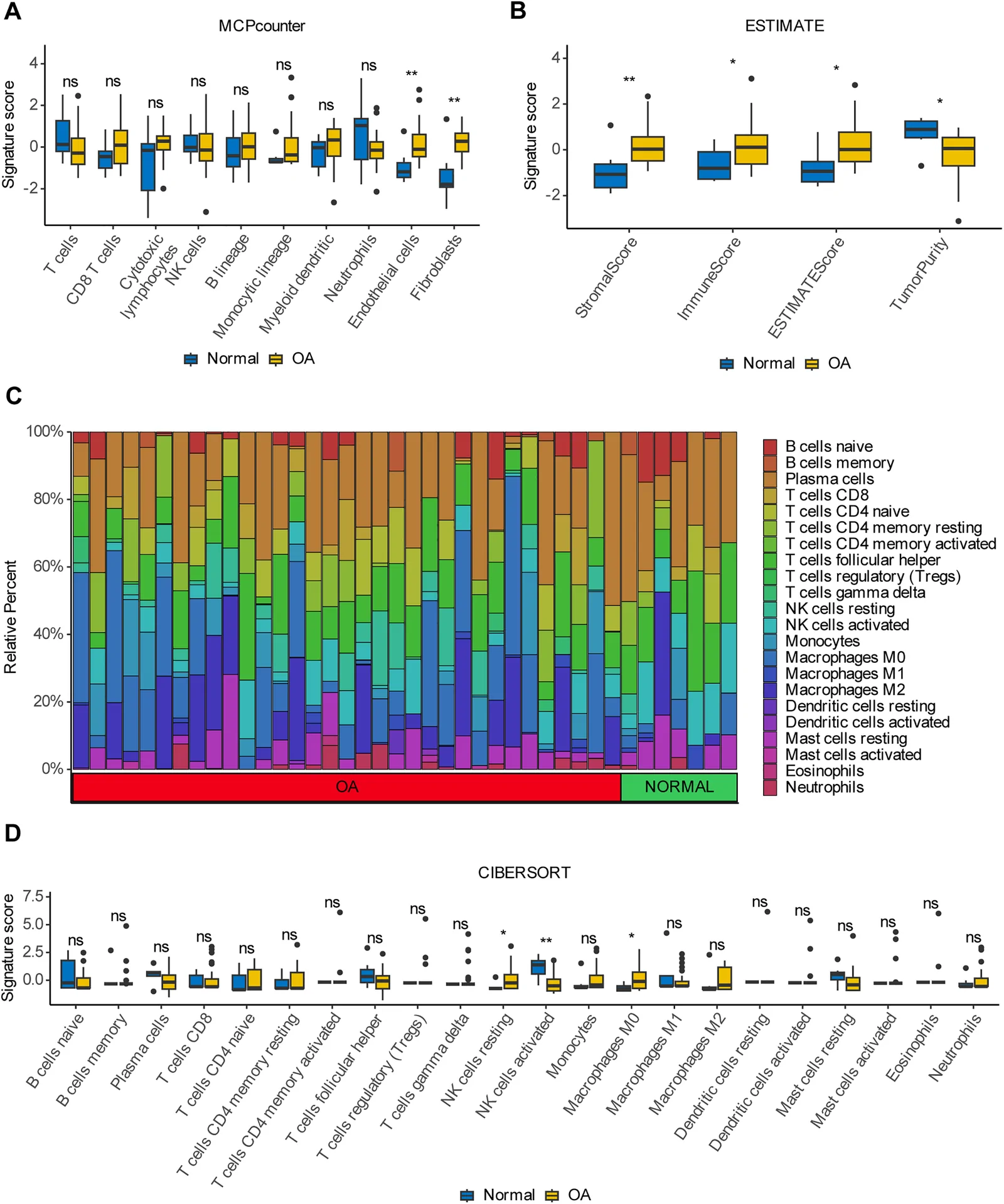
Immune analysis. (A,B) Immune cell subset enrichment scores were computed with the IOBR package by integrating MCP-counter and ESTIMATE, and compared between OA (yellow) and normal (blue) samples. Group differences were evaluated using the two-sided Wilcoxon rank-sum test (Mann–Whitney U). *p < 0.05, **p < 0.01, ***p < 0.001.(C) CIBERSORT was used to evaluate the relative proportions of 22 immune cell types in OA versus normal samples. (D) Bar charts illustrate the distribution of these immune subsets, with Wilcoxon test applied to assess group differences (*p < 0.05, **p < 0.01, ***p < 0.001).

**Fig 11:**
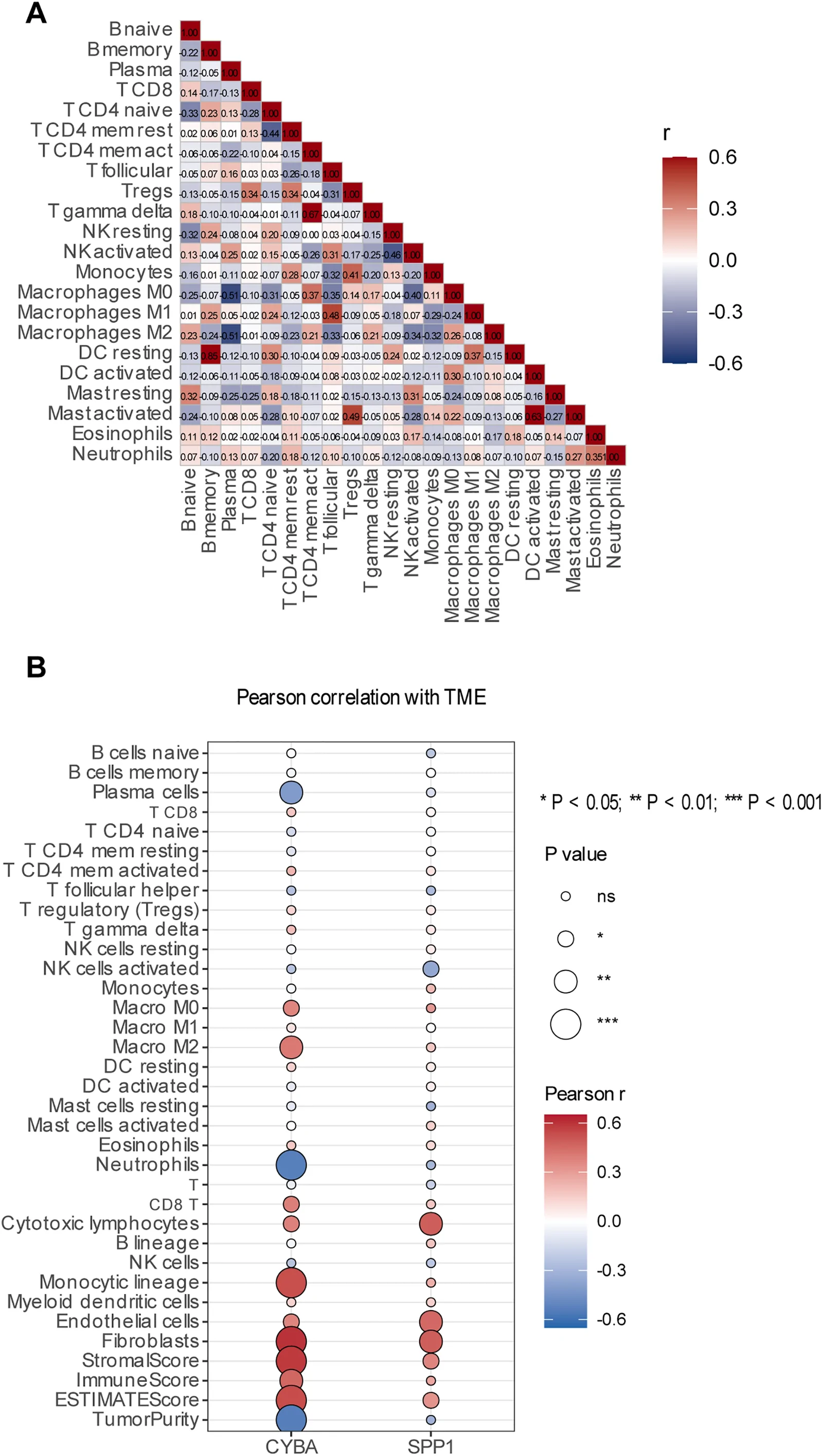
Correlations among immune cell subsets and associations of CYBA and SPP1 with the OA immune microenvironment. (A) Correlation heatmap of 22 immune cell populations, where red denotes positive correlations, blue indicates negative ones, and color depth represents correlation strength. (B) Bubble plot showing Spearman correlations between *CYBA*/*SPP1* expression and immune features. Red denotes positive correlations, blue negative; bubble size reflects correlation strength, and color intensity indicates significance.

Correlation analysis of the chemokine and TNF superfamilies showed significant associations with *CYBA*, *SPP1*, and RiskScore (Fig 12A). These findings suggest that oxidative stress-related genes may participate in OA-related inflammatory regulation through chemokine- and TNF-mediated signaling pathways.

**Fig 12:**
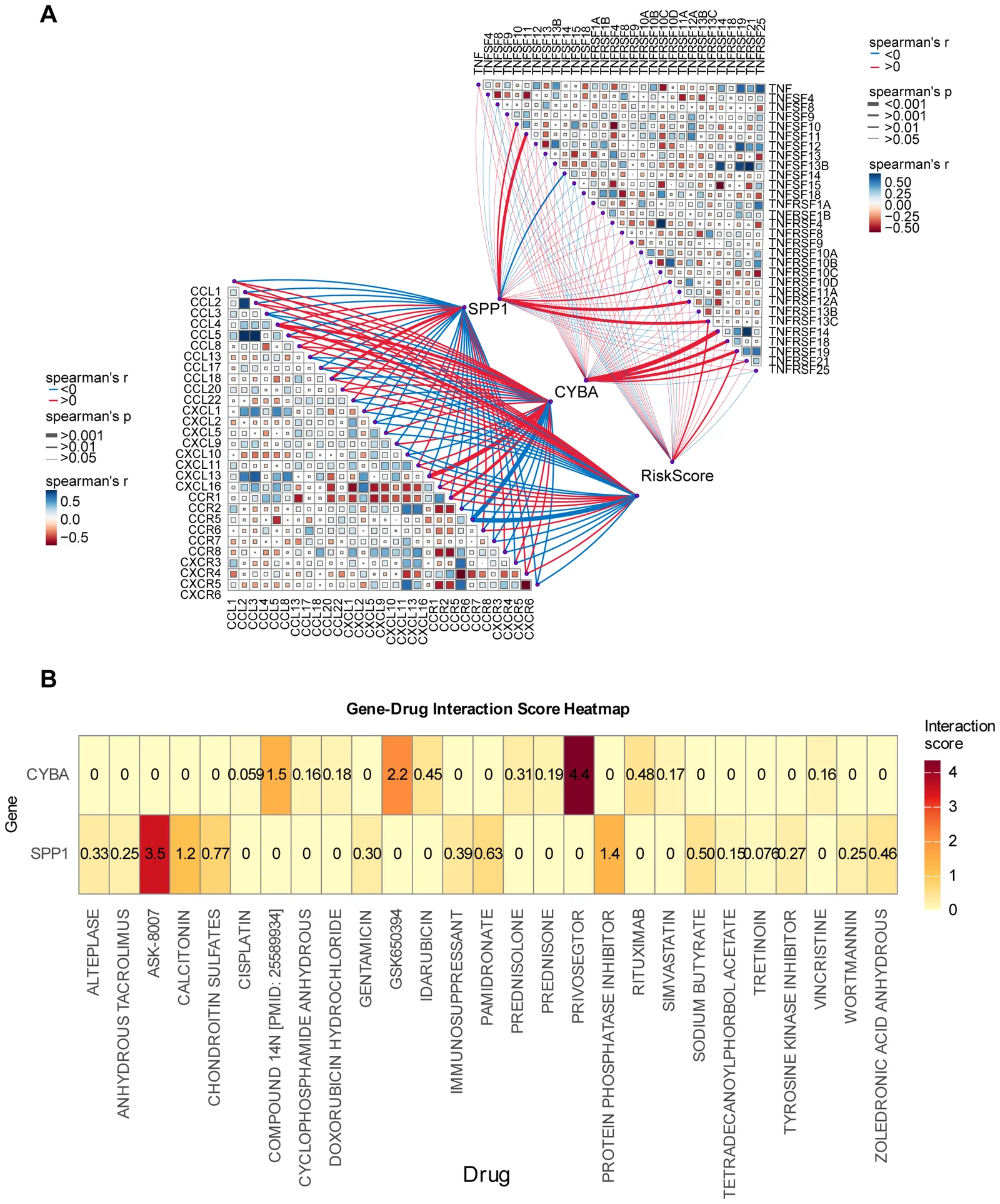
Associations of CYBA, SPP1, and RiskScore with chemokine and TNF superfamily members and predicted drug interactions. (A) Correlation network of *CYBA*, *SPP1*, and RiskScore with chemokines and TNF family members, where red/blue edges represent positive/negative associations and line width corresponds to correlation magnitude. (B) Heatmap of *CYBA* and *SPP1* drug interaction scores based on the Drug–Gene Interaction database (DGIdb), with colors from light to dark indicating increasing interaction scores.

To identify potential pharmacological interventions, drug-gene interaction scores between *CYBA*, *SPP1*, and known drugs were retrieved from the drug-gene interaction database (DGIdb). The interaction profiles were visualized as a heatmap (Fig 12B), highlighting candidate compounds for targeted therapy in OA.

### Validation of core gene expression by qPCR and Western Blot (WB)

Core gene expression was verified by quantitative real-time PCR (qPCR). In H_2_O_2-_induced OA chondrocytes, *MMP13* was markedly upregulated, while Col2a1 was downregulated, confirming successful OA model establishment. Compared with normal chondrocytes, the mRNA levels of CYBA and SPP**1** were markedly increased (Fig 13A). WB analysis further validated the protein expression of these core genes (Fig 13B), showing that both *CYBA* and *SPP1* protein levels were significantly elevated in H_2_O_2-_induced OA cells. The original, uncropped full-length Western blot images with visible edges and molecular weight markers are provided in the Supplementary Materials (S1 File).

**Fig 13:**
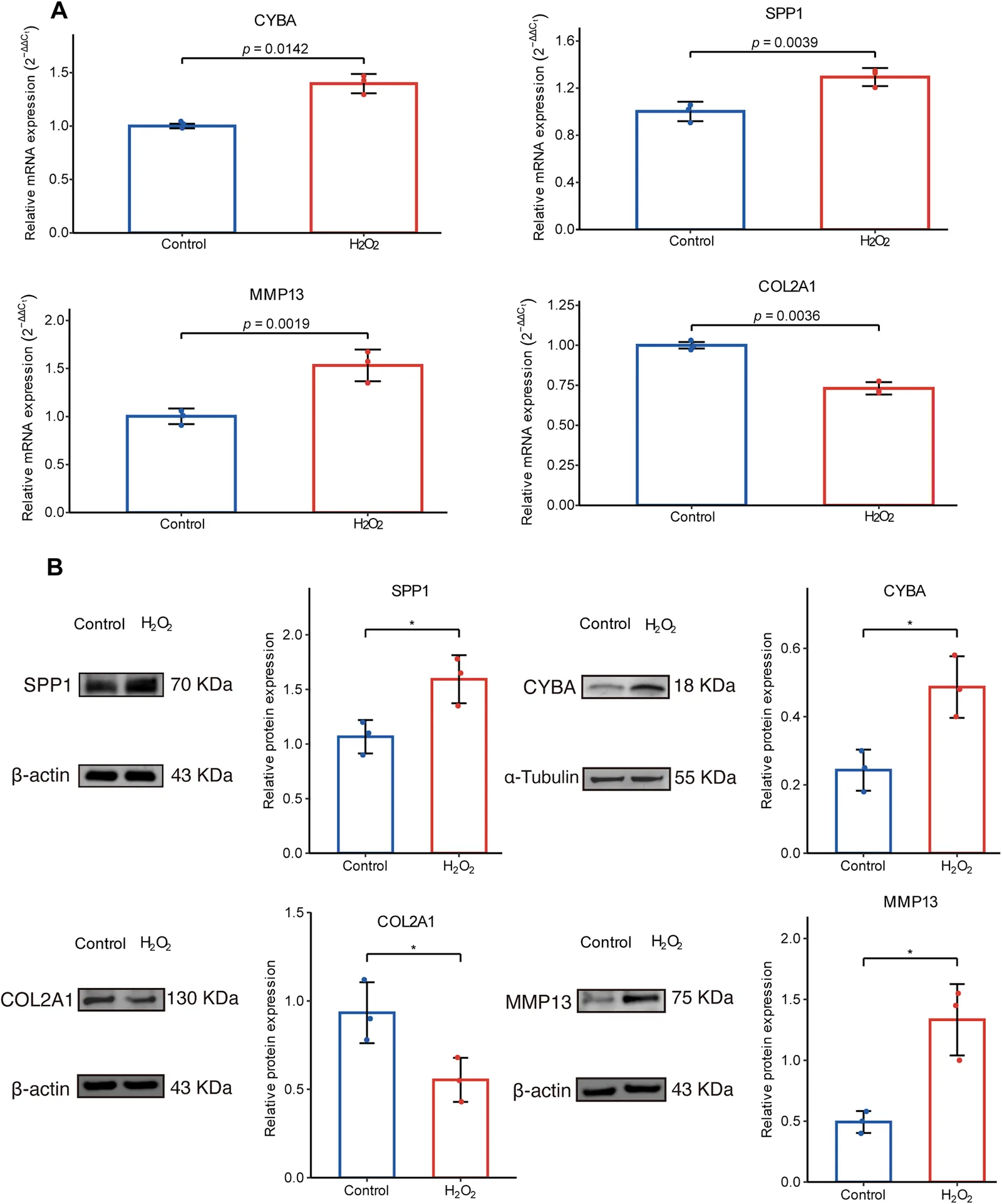
Validation of core genes and cartilage-related proteins in an H_2_O_2_-induced inflammatory model. **(A)** qRT-PCR analysis of *Cyba*, *Spp1*, *Mmp13*, and *Col2a1*. Individual points represent biological replicates (n = 3), and bars show mean ± SD. Exact *P* values are indicated. **(B)** Representative western blots and densitometric analysis of *CYBA*, *SPP1*, *MMP13*, and *COL2A1*. β-actin or α-tubulin served as loading controls. Individual points represent biological replicates (n = 3), and bars show mean ± SD, *P < 0.05.

## Discussion

Previous studies have demonstrated that disruption of mitochondrial redox homeostasis and excessive accumulation of ROS play critical roles in the development of KOA [16]. Mechanical overload and aging increase mitochondrial superoxide production while reducing *SOD2* expression in chondrocytes. This redox imbalance impairs mitochondrial function, promotes telomere shortening, reduces proliferative capacity, and compromises glycosaminoglycan synthesis, ultimately accelerating chondrocyte senescence and apoptosis [17,18]. Moreover, matrix degradation products and pro-inflammatory cytokines released by degenerative chondrocytes can activate synovial cells, triggering synovial inflammation [19]. In turn, chronic synovial inflammation aggravates oxidative stress, mitochondrial DNA damage, tissue hypoxia, and immune activation, establishing a self-reinforcing cycle of joint injury [20]. Together, these processes link oxidative stress to cartilage degeneration and synovial inflammation in KOA**, supporting its potential relevance as a therapeutic target.**

Single-cell clustering identified 11 functionally heterogeneous chondrocyte subpopulations. InfC and ProFC were exclusively detected in OA samples, accounting for 0.38% and 0.20% of chondrocytes, respectively. Previous studies reported proportions of approximately 1.2% and 1%, indicating that InfC and ProFC represent rare OA**-associated subsets** [13,14]. GO and KEGG enrichment and AUCell analyses showed that InfC was enriched in chemokine signaling, inflammatory factor expression, and immune cell migration. Consistent with these findings, previous studies have reported that InfC expresses inflammatory markers and is preferentially localized to the middle zone (MZ) of OA cartilage [13]. ProFC exhibited OA-specific expression patterns, heightened oxidative stress activity, and enrichment in cell cycle regulation, cellular senescence, DNA repair, and ECM remodeling during OA progression, suggesting its involvement in proliferative and maladaptive repair responses [14].

Analysis of cell-cell communication revealed marked increases in both the frequency and strength of intercellular signaling among chondrocytes in OA, consistent with previous reports [21]. The COLLAGEN and *FN****1*** signaling pathways exhibited widespread ligand-receptor activity, with **Pro**FC acting as a major signaling hub. *FN1* is a multifunctional glycoprotein involved in cell-matrix adhesion complexes and ECM organization [22,23]. Inflammatory cleavage of *FN1* can generate fragments that impair chondrocyte differentiation and promote cartilage degradation [24]. Moreover, Collagen-Integrins, Fibronectin-Integrins, and Tenascin-Syndecan **interactions mediate communication** among chondrocytes, synoviocytes, and meniscal cells [25]. Together, these findings suggest that the ProFC subtype functions as a pivotal hub in OA cell-cell communication, underscoring its potential role in disease pathogenesis and warranting further in-depth investigation.

Monocle pseudotime analysis revealed that chondrocyte subpopulations associated with fibrosis and inflammation, including ProFC and InfC, were predominantly enriched at the terminal end of the trajectory. This pattern suggests that, under the influence of the OA microenvironment, these cells may preferentially differentiate toward pathological terminal states, thereby contributing to tissue structural damage and the persistence of chronic inflammatory processes.

Integration of machine learning and bioinformatics analyses identified CYBA and SPP*1* as candidate oxidative stress-related genes. The resulting two-gene model showed high discrimination in the training cohort and retained moderate discriminatory ability in the independent validation cohort, while in vitro assays supported increased expression under oxidative stress conditions.

p22^phox was first identified in 1987 within the plasma membrane of human neutrophils [26]. It serves as a critical structural subunit of the NADPH oxidase complex, functioning alongside catalytic subunits such as *NOX1*, *NOX2*, *NOX3*, and *NOX4* to mediate the generation of ROS [27]. *CYBA* is highly expressed in hematopoietic tissues, including bone marrow, blood, and leukocytes. During phagocyte activation, it rapidly induces ROS production (e.g., superoxide anion) to eliminate pathogens, playing a central role in innate immune defense [28]. Mutations in *CYBA* can impair NADPH oxidase function, leading to chronic granulomatous disease (CGD), a prototypical immunodeficiency disorder [29]. In human chondrocytes, *NOX4* is the predominant NOX isoform. Pro-inflammatory cytokines such as IL-1β can activate the *NOX4*-p22^phox**-**ROS axis, which amplifies signaling via a ROS-mediated positive feedback loop, thereby promoting catabolic activity in chondrocytes [30]. Pharmacological inhibition of p22^phox suppresses NADPH oxidase activity, reduces oxidative stress, and mitigates the severity of RA [31]. Although the contribution of CYBA variants to OA susceptibility remains uncertain [32], **t**he role of p22phox in ROS **production supports its potential involvement in** OA**-related oxidative damage**. Accordingly, targeting p22phox or its associated signaling pathways may represent a potential therapeutic strategy for OA and other inflammatory diseases.

*SPP****1***, also known as osteopontin (OPN), is a phosphorylated glycoprotein expressed by lymphocytes, chondrocytes, and synovial cells [33,34]. Numerous studies have reported OPN expression in OA cartilage across different disease stages, with levels positively correlating with disease severity. Elevated OPN promotes synovial angiogenesis and chondrocyte apoptosis, thereby contributing to joint destruction in OA [35,36]. Mechanistically, OPN interacts with receptors such as *CD44* and integrin αvβ3 to upregulate hyaluronan synthase 1 (*HAS1*) and hyaluronic acid (HA), facilitating synovitis and ECM remodeling [37]. Additionally, OPN has been shown to induce MMP**-**13 expression while downregulating *COL****2A1***, promoting cartilage degradation [38]. Interestingly, OPN exhibits dual functionality in OA. On the one hand, its upregulation can activate the PI3K**/**AKT**/m**TOR pathway, promoting cartilage degradation, bone remodeling, and inflammation, thereby accelerating OA progression [39]. On the other hand, evidence from certain studies suggests that OPN may exert protective effects via activation of the PI3K signaling cascade, suppressing chondrocyte apoptosis and slowing ECM degradation [40]. These findings indicate that OPN can mediate both “adaptive protection” and “pathological exacerbation” in OA. It is hypothesized that initial OPN upregulation represents a defensive response to inflammation and catabolic stress; however, this protective mechanism may become dysregulated during later stages of cartilage degeneration, contributing to disease progression [41]. Collectively, *SPP****1*** may act as a key regulatory factor with dynamic roles in OA and may represent a potential therapeutic target.

OA is increasingly recognized as a disease involving innate immune activation and chronic inflammation rather than cartilage degeneration alone [42]. The results of this study indicate that oxidative stress–related core genes and their regulatory networks are closely associated with immune cell infiltration patterns and multiple immune-related features. These findings suggest that redox imbalance may contribute to immune microenvironment remodeling by modulating inflammatory cell infiltration and immune signaling, thereby playing an important role in the initiation and progression of OA.

Although InfC and ProFC were detected only in OA samples in the present dataset, their low abundance warrants cautious interpretation. Differences in sample composition, disease stage, cartilage sampling region, tissue dissociation efficiency, sequencing depth, and analytical methods may partly explain the variation in their reported frequencies and potential underestimation. Nevertheless, both subsets showed consistent enrichment in oxidative stress, inflammatory, immune, and extracellular matrix remodeling programs, supported by AUCell scoring, pseudotime distribution, and cell–cell communication analyses. Thus, despite their rarity, InfC and ProFC may have biological relevance in OA, consistent with previous single-cell studies showing that populations comprising less than 1% of total cells can provide important mechanistic insights [43,44]. *CYBA* and *SPP1* are candidate molecules linking oxidative stress, ECM alterations, and immune remodeling, and a diagnostic model based on these genes performed well across multiple cohorts with in vitro support. Given the limited sample sizes, incomplete clinical covariates, and heterogeneity of public datasets, future studies should validate these findings in large, independent cohorts with well-defined clinical phenotypes and integrate spatial transcriptomics with functional perturbation experiments to clarify causality and assess their clinical translational potential.

## Conclusions

This study identified ProFC and InfC as OA-associated chondrocyte subpopulations characterized by elevated oxidative stress and enhanced inflammatory and immune activity. CYBA and SPP*1* showed potential as candidate diagnostic biomarkers. These findings provide insight into oxidative stress-related cellular heterogeneity and immune-inflammatory mechanisms in OA but require validation in larger independent cohorts and functional studies before clinical translation.

## Supporting information

S1 Table Clinical characteristics of the bulk RNA-seq cohorts used for model training and validation. Clinical information available from the original GEO records and associated publications for the GSE57218 training cohort and GSE114007 validation cohort is summarized.
(XLSX)

S1 Dataset Oxidative stress-related genes used for AUCell scoring. This dataset contains the 1,491 oxidative stress-related genes obtained from GeneCards with a relevance score greater than 8.
(CSV)

S2 Dataset Differentially expressed genes associated with oxidative stress scores. The dataset includes 658 differentially expressed genes identified between high- and low-AUCell score groups using Seurat analysis (logFC > 0.25, *P* < 0.05).
(CSV)

S3 Dataset hdWGCNA-derived hub genes and integrated oxidative stress-related genes. The dataset includes hub genes identified from the M2 and M3 modules of hdWGCNA and the overlapping genes obtained through integration with oxidative stress-related differentially expressed genes.
(CSV)

S4 Dataset Complete outputs of machine-learning feature selection and interpretation analyses. This dataset contains the complete results of LASSO regression, SVM-RFE, and XGBoost with SHAP analyses, including selected genes, feature rankings, and model interpretation results.
(CSV)

S1 File Original uncropped and unadjusted images underlying the western blot results. This file contains the original uncropped and unadjusted images underlying all western blot results presented in the main manuscript and Supporting Information. Each image is labeled with the corresponding figure panel, target protein, molecular weight, sample identity, lane order, loading control, and relevant image acquisition information.
(PDF)

S1 Fig Calibration curves and diagnostic performance of the diagnostic model. Calibration curves and diagnostic performance metrics, including sensitivity, specificity, PPV, and NPV with 95% confidence intervals, are shown for the training cohort (GSE57218) and validation cohort (GSE114007).
(TIF)

## References

[1] Zhang B-F, Liu L, Xu S-L, Yang Z. Global, regional and national burden of knee osteoarthritis 1990-2021: A systematic analysis of the Global Burden of Disease study 2021. Arch Gerontol Geriatr. 2025;136:105867. doi: 10.1016/j.archger.2025.105867 40403596

[2] Musumeci G, Aiello FC, Szychlinska MA, Di Rosa M, Castrogiovanni P, Mobasheri A. Osteoarthritis in the XXIst century: Risk factors and behaviours that influence disease onset and progression. Int J Mol Sci. 2015;16(3):6093–112. doi: 10.3390/ijms16036093 25785564 PMC4394521

[3] Quinn RH, Murray JN, Pezold R, Sevarino KS. Surgical Management of osteoarthritis of the knee. J Am Acad Orthop Surg. 2018;26(9):e191–3. doi: 10.5435/JAAOS-D-17-00424 29688919

[4] Regan E, Flannelly J, Bowler R, Tran K, Nicks M, Carbone BD, et al. Extracellular superoxide dismutase and oxidant damage in osteoarthritis. Arthritis Rheum. 2005;52(11):3479–91. doi: 10.1002/art.21387 16255039 PMC2755499

[5] Bolduc JA, Collins JA, Loeser RF. Reactive oxygen species, aging and articular cartilage homeostasis. Free Radic Biol Med. 2019;132:73–82. doi: 10.1016/j.freeradbiomed.2018.08.038 30176344 PMC6342625

[6] Annesley SJ, Fisher PR. Mitochondria in health and disease. Cells. 2019;8(7):680. doi: 10.3390/cells8070680 31284394 PMC6678092

[7] Ulfig A, Jakob U. Cellular oxidants and the proteostasis network: balance between activation and destruction. Trends Biochem Sci. 2024;49(9):761–74. doi: 10.1016/j.tibs.2024.07.001 39168791 PMC11731897

[8] Moniruzzaman M, Ghosal I, Das D, Chakraborty SB. Melatonin ameliorates H2O2-induced oxidative stress through modulation of Erk/Akt/NFkB pathway. Biol Res. 2018;51(1):17. doi: 10.1186/s40659-018-0168-5 29891016 PMC5996524

[9] Angajala A, Lim S, Phillips JB, Kim J-H, Yates C, You Z, et al. Diverse roles of mitochondria in immune responses: Novel insights into immuno-metabolism. Front Immunol. 2018;9:1605. doi: 10.3389/fimmu.2018.01605 30050539 PMC6052888

[10] Khozyainova AA, Valyaeva AA, Arbatsky MS, Isaev SV, Iamshchikov PS, Volchkov EV, et al. Complex analysis of single-cell RNA sequencing data. Biochemistry (Mosc). 2023;88(2):231–52. doi: 10.1134/S0006297923020074 37072324 PMC10000364

[11] Ji Q, Zheng Y, Zhang G, Hu Y, Fan X, Hou Y, et al. Single-cell RNA-seq analysis reveals the progression of human osteoarthritis. Ann Rheum Dis. 2019;78(1):100–10. doi: 10.1136/annrheumdis-2017-212863 30026257 PMC6317448

[12] Chou C-H, Jain V, Gibson J, Attarian DE, Haraden CA, Yohn CB, et al. Synovial cell cross-talk with cartilage plays a major role in the pathogenesis of osteoarthritis. Sci Rep. 2020;10(1):10868. doi: 10.1038/s41598-020-67730-y 32616761 PMC7331607

[13] Fan Y, Bian X, Meng X, Li L, Fu L, Zhang Y, et al. Unveiling inflammatory and prehypertrophic cell populations as key contributors to knee cartilage degeneration in osteoarthritis using multi-omics data integration. Ann Rheum Dis. 2024;83(7):926–44. doi: 10.1136/ard-2023-224420 38325908 PMC11187367

[14] Huang C, Zeng B, Zhou B, Chen G, Zhang Q, Hou W, et al. Single-cell transcriptomic analysis of chondrocytes in cartilage and pathogenesis of osteoarthritis. Genes Dis. 2024;12(2):101241. doi: 10.1016/j.gendis.2024.101241 39759119 PMC11697194

[15] Zeng D, Ye Z, Shen R, Yu G, Wu J, Xiong Y, et al. IOBR: Multi-omics immuno-oncology biological research to decode tumor microenvironment and signatures. Front Immunol. 2021;12:687975. doi: 10.3389/fimmu.2021.687975 34276676 PMC8283787

[16] Scott JL, Gabrielides C, Davidson RK, Swingler TE, Clark IM, Wallis GA, et al. Superoxide dismutase downregulation in osteoarthritis progression and end-stage disease. Ann Rheum Dis. 2010;69(8):1502–10. doi: 10.1136/ard.2009.119966 20511611 PMC3789136

[17] Neri S, Guidotti S, Panichi V, Minguzzi M, Cattini L, Platano D, et al. IKKα affects the susceptibility of primary human osteoarthritis chondrocytes to oxidative stress-induced DNA damage by tuning autophagy. Free Radic Biol Med. 2024;225:726–40. doi: 10.1016/j.freeradbiomed.2024.10.299 39461484

[18] Koike M, Nojiri H, Ozawa Y, Watanabe K, Muramatsu Y, Kaneko H, et al. Mechanical overloading causes mitochondrial superoxide and SOD2 imbalance in chondrocytes resulting in cartilage degeneration. Sci Rep. 2015;5:11722. doi: 10.1038/srep11722 26108578 PMC4480010

[19] Favero M, El-Hadi H, Belluzzi E, Granzotto M, Porzionato A, Sarasin G, et al. Infrapatellar fat pad features in osteoarthritis: a histopathological and molecular study. Rheumatology (Oxford). 2017;56(10):1784–93. doi: 10.1093/rheumatology/kex287 28957567

[20] Balogh E, Veale DJ, McGarry T, Orr C, Szekanecz Z, Ng C-T, et al. Oxidative stress impairs energy metabolism in primary cells and synovial tissue of patients with rheumatoid arthritis. Arthritis Res Ther. 2018;20(1):95. doi: 10.1186/s13075-018-1592-1 29843785 PMC5972404

[21] Kang X, Zhang K, Wang Y, Zhao Y, Lu Y. Single-cell RNA sequencing analysis of human chondrocytes reveals cell-cell communication alterations mediated by interactive signaling pathways in osteoarthritis. Front Cell Dev Biol. 2023;11:1099287. doi: 10.3389/fcell.2023.1099287 37082621 PMC10112522

[22] Shiozawa K, Shiozawa S, Shimizu S, Fujita T. Fibronectin on the surface of articular cartilage in rheumatoid arthritis. Arthritis Rheum. 1984;27(6):615–22. doi: 10.1002/art.1780270603 6203543

[23] Chevalier X. Fibronectin, cartilage, and osteoarthritis. Semin Arthritis Rheum. 1993;22(5):307–18. doi: 10.1016/s0049-0172(05)80010-1 8511595

[24] Aladal M, You W, Huang R, Huang J, Deng Z, Duan L, et al. Insights into the implementation of Fibronectin 1 in the cartilage tissue engineering. Biomed Pharmacother. 2022;148:112782. doi: 10.1016/j.biopha.2022.112782 35248846

[25] Wang M, Tan G, Jiang H, Liu A, Wu R, Li J, et al. Molecular crosstalk between articular cartilage, meniscus, synovium, and subchondral bone in osteoarthritis. Bone Joint Res. 2022;11(12):862–72. doi: 10.1302/2046-3758.1112.BJR-2022-0215.R1 36464496 PMC9792876

[26] Parkos CA, Allen RA, Cochrane CG, Jesaitis AJ. Purified cytochrome b from human granulocyte plasma membrane is comprised of two polypeptides with relative molecular weights of 91,000 and 22,000. J Clin Invest. 1987;80(3):732–42. doi: 10.1172/JCI113128 3305576 PMC442297

[27] Ambasta RK, Kumar P, Griendling KK, Schmidt HHHW, Busse R, Brandes RP. Direct interaction of the novel Nox proteins with p22phox is required for the formation of a functionally active NADPH oxidase. J Biol Chem. 2004;279(44):45935–41. doi: 10.1074/jbc.M406486200 15322091

[28] Beaumel S, Grunwald D, Fieschi F, Stasia MJ. Identification of NOX2 regions for normal biosynthesis of cytochrome b558 in phagocytes highlighting essential residues for p22phox binding. Biochem J. 2014;464(3):425–37. doi: 10.1042/BJ20140555 25252997

[29] Bakri FG, Martel C, Khuri-Bulos N, Mahafzah A, El-Khateeb MS, Al-Wahadneh AM, et al. First report of clinical, functional, and molecular investigation of chronic granulomatous disease in nine Jordanian families. J Clin Immunol. 2009;29(2):215–30. doi: 10.1007/s10875-008-9243-y 18773283

[30] Rousset F, Hazane-Puch F, Pinosa C, Nguyen MVC, Grange L, Soldini A, et al. IL-1beta mediates MMP secretion and IL-1beta neosynthesis via upregulation of p22(phox) and NOX4 activity in human articular chondrocytes. Osteoarthritis Cartilage. 2015;23(11):1972–80. doi: 10.1016/j.joca.2015.02.167 26521743

[31] Kim Y-R, Kim J-S, Gu S-J, Jo S, Kim S, Young Kim S, et al. Identification of highly potent and selective inhibitor, TIPTP, of the p22phox-Rubicon axis as a therapeutic agent for rheumatoid arthritis. Sci Rep. 2020;10(1):4570. doi: 10.1038/s41598-020-61630-x 32165681 PMC7067850

[32] Lepetsos P, Pampanos A, Lallos S, Kanavakis E, Korres D, Papavassiliou AG, et al. Association of NADPH oxidase p22phox gene C242T, A640G and -930A/G polymorphisms with primary knee osteoarthritis in the Greek population. Mol Biol Rep. 2013;40(9):5491–9. doi: 10.1007/s11033-013-2649-5 23922196

[33] Xie Y, Sakatsume M, Nishi S, Narita I, Arakawa M, Gejyo F. Expression, roles, receptors, and regulation of osteopontin in the kidney. Kidney Int. 2001;60(5):1645–57. 10.1046/j.1523-1755.2001.00032.x 11703581

[34] Hasegawa M, Nakoshi Y, Iino T, Sudo A, Segawa T, Maeda M, et al. Thrombin-cleaved osteopontin in synovial fluid of subjects with rheumatoid arthritis. J Rheumatol. 2009;36(2):240–5. doi: 10.3899/jrheum.080753 19208558

[35] Gao SG, Li KH, Zeng KB, Tu M, Xu M, Lei GH. Elevated osteopontin level of synovial fluid and articular cartilage is associated with disease severity in knee osteoarthritis patients. Osteoarthritis Cartilage. 2010;18(1):82–7. doi: 10.1016/j.joca.2009.07.009 19747583

[36] Yumoto K, Ishijima M, Rittling SR, Tsuji K, Tsuchiya Y, Kon S, et al. Osteopontin deficiency protects joints against destruction in anti-type II collagen antibody-induced arthritis in mice. Proc Natl Acad Sci U S A. 2002;99(7):4556–61. doi: 10.1073/pnas.052523599 11930008 PMC123686

[37] Zhang F-J, Gao S-G, Cheng L, Tian J, Xu W-S, Luo W, et al. The effect of hyaluronic acid on osteopontin and CD44 mRNA of fibroblast-like synoviocytes in patients with osteoarthritis of the knee. Rheumatol Int. 2013;33(1):79–83. doi: 10.1007/s00296-011-2339-3 22218639

[38] Zhang Y, Wang T, Cao Y. Osteopontin can decrease the expression of Col-II and COMP in cartilage cells in vitro. Int J Clin Exp Med. 2015;8(2):2254–60. 25932159 PMC4402806

[39] Lin C, Chen Z, Guo D, Zhou L, Lin S, Li C, et al. Increased expression of osteopontin in subchondral bone promotes bone turnover and remodeling, and accelerates the progression of OA in a mouse model. Aging (Albany NY). 2022;14(1):253–71. doi: 10.18632/aging.203707 34982732 PMC8791213

[40] Liu Q, Zeng H, Yuan Y, Wang Z, Wu Z, Luo W. Osteopontin inhibits osteoarthritis progression via the OPN/CD44/PI3K signal axis. Genes Dis. 2020;9(1):128–39. doi: 10.1016/j.gendis.2020.06.006 35005113 PMC8720673

[41] Sun K, Luo J, Guo J, Yao X, Jing X, Guo F. The PI3K/AKT/mTOR signaling pathway in osteoarthritis: a narrative review. Osteoarthritis Cartilage. 2020;28(4):400–9. doi: 10.1016/j.joca.2020.02.027 32081707

[42] Liu M, Wu C, Wu C, Zhou Z, Fang R, Liu C, et al. Immune cells differentiation in osteoarthritic cartilage damage: friends or foes?. Front Immunol. 2025;16:1545284. doi: 10.3389/fimmu.2025.1545284 40201177 PMC11975574

[43] Grün D, Lyubimova A, Kester L, Wiebrands K, Basak O, Sasaki N, et al. Single-cell messenger RNA sequencing reveals rare intestinal cell types. Nature. 2015;525(7568):251–5. doi: 10.1038/nature14966 26287467

[44] Jiang L, Chen H, Pinello L, Yuan G-C. GiniClust: detecting rare cell types from single-cell gene expression data with Gini index. Genome Biol. 2016;17(1):144. doi: 10.1186/s13059-016-1010-4 27368803 PMC4930624

