# Supplementary material for: Oxidative stress-driven cellular heterogeneity in Knee Osteoarthritis: scRNA-seq pinpoints SPP1 and CYBA for diagnostic development
Source: PLoS One. 2026 Oct 27;21(10):e0360537. doi: 10.1371/journal.pone.0360537 (PMC13712592; doi:10.1371/journal.pone.0360537)

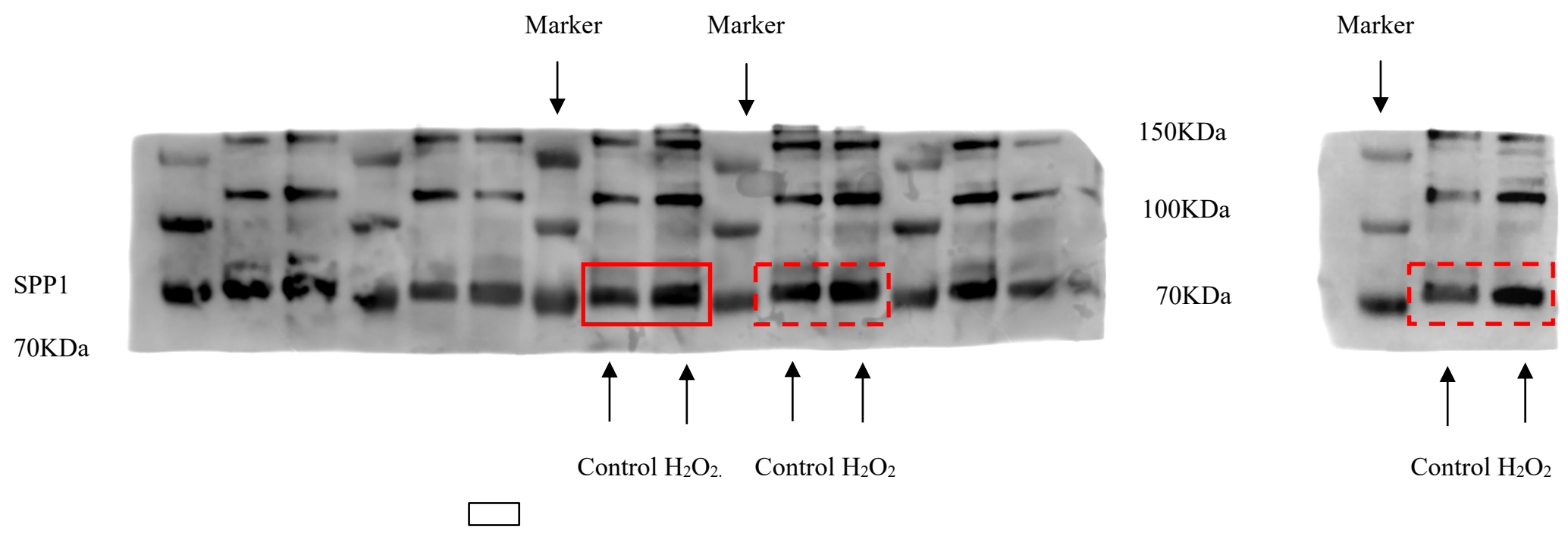
Marker
Marker
Marker
150KDa
100KDa
70KDa
SPP1
70KDa
Control H_2O_2.
Control H_2O_2
Control H_2O_2

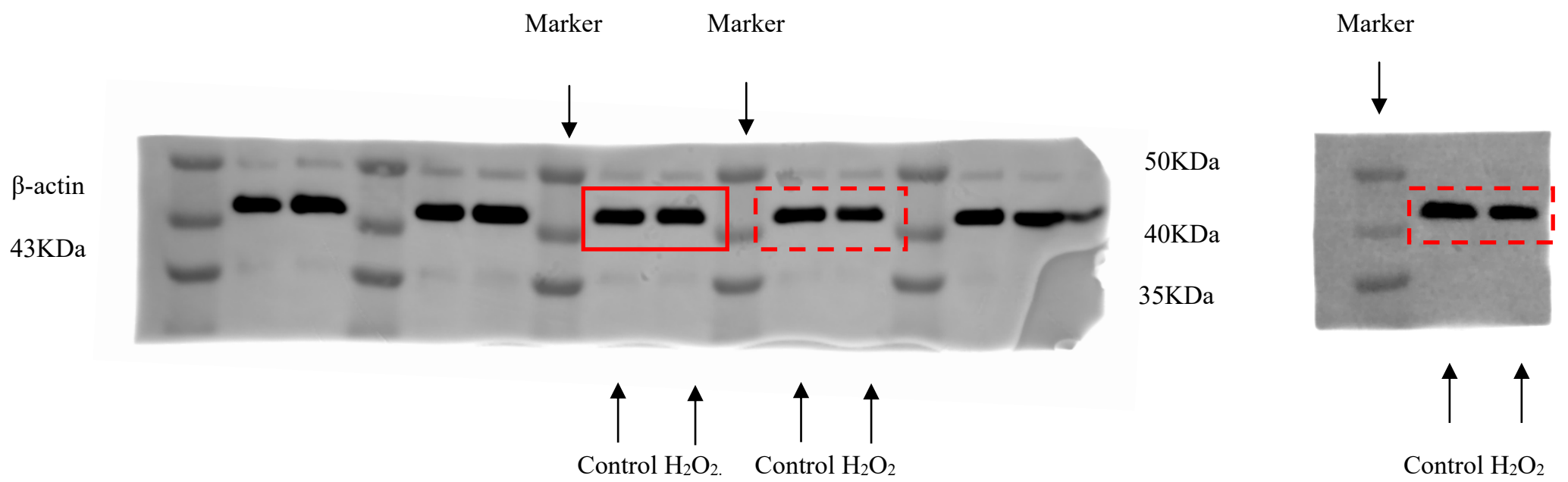
Marker
Marker
Marker
β-actin
43KDa
50KDa
40KDa
35KDa
Control H_2O_2.
Control H_2O_2
Control H_2O_2

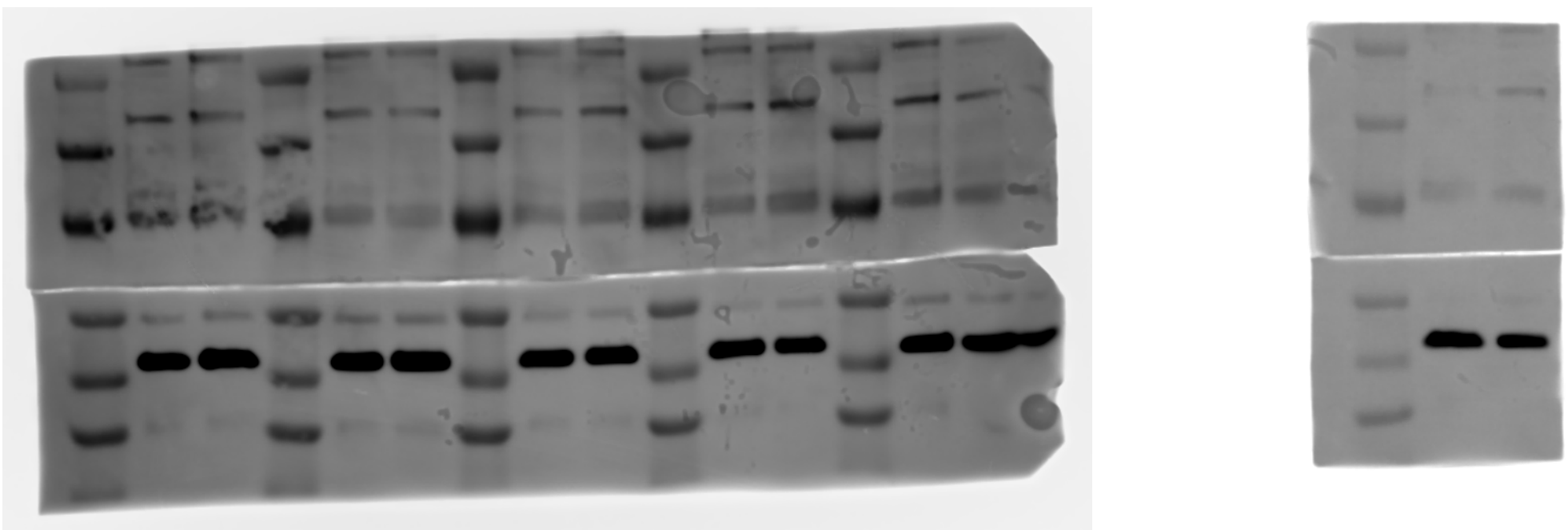

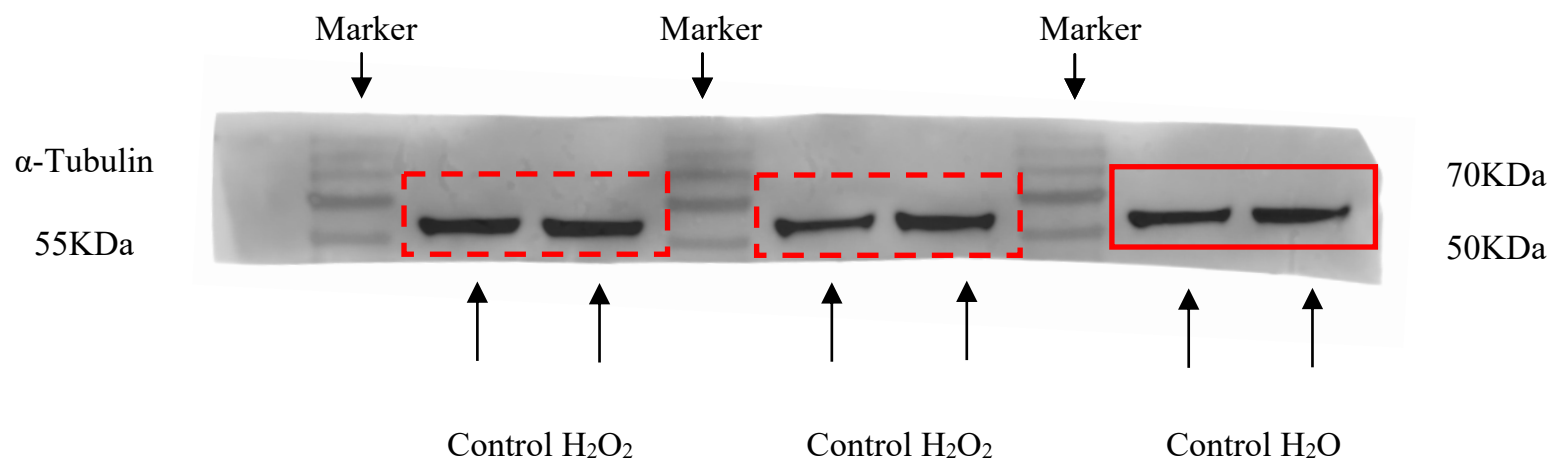
Marker
Marker
Marker
α-Tubulin
55KDa
70KDa
50KDa
Control H_2O_2
Control H_2O_2
Control H_2O

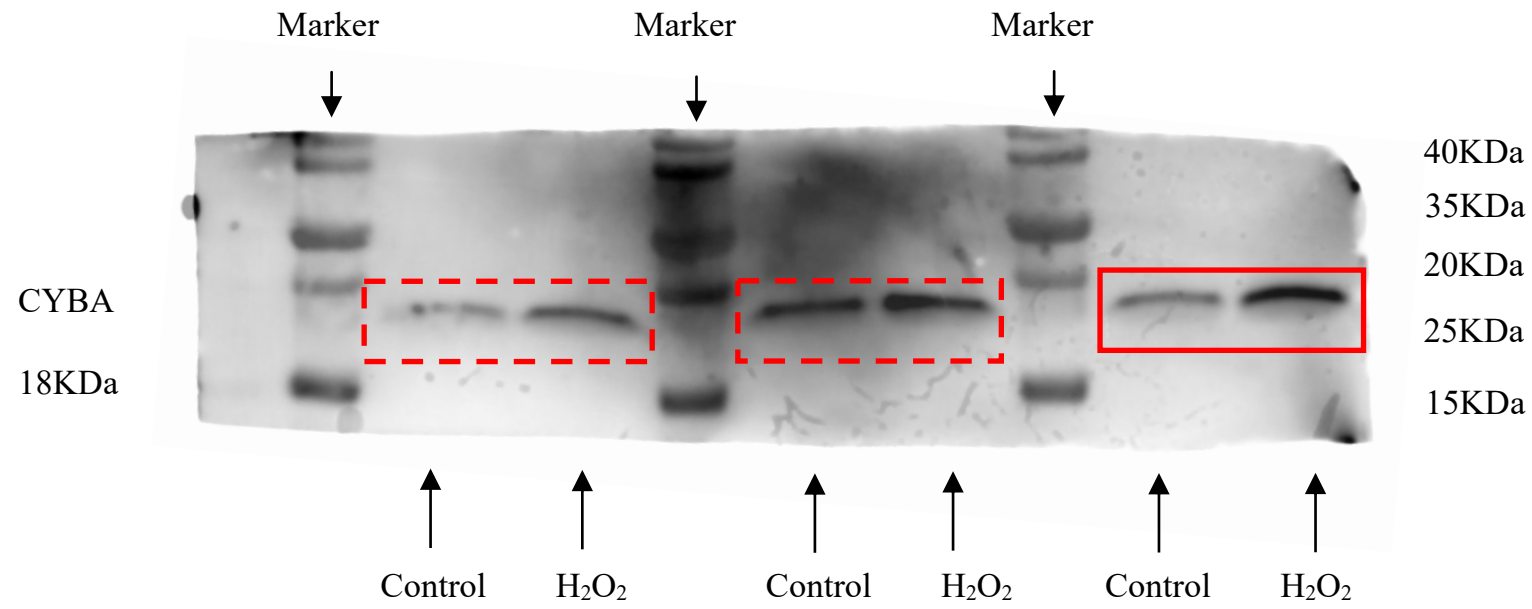
Marker
Marker
Marker
40KDa
35KDa
20KDa
CYBA
25KDa
18KDa
15KDa
Control
H_2O_2
Control
H_2O_2
Control
H_2O_2

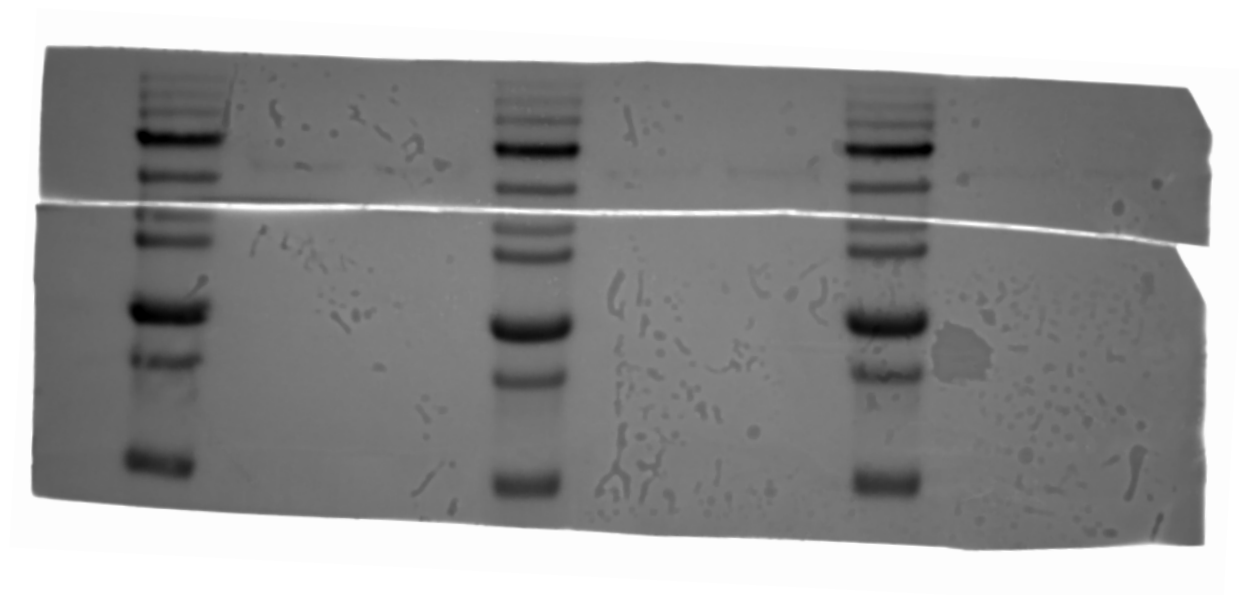

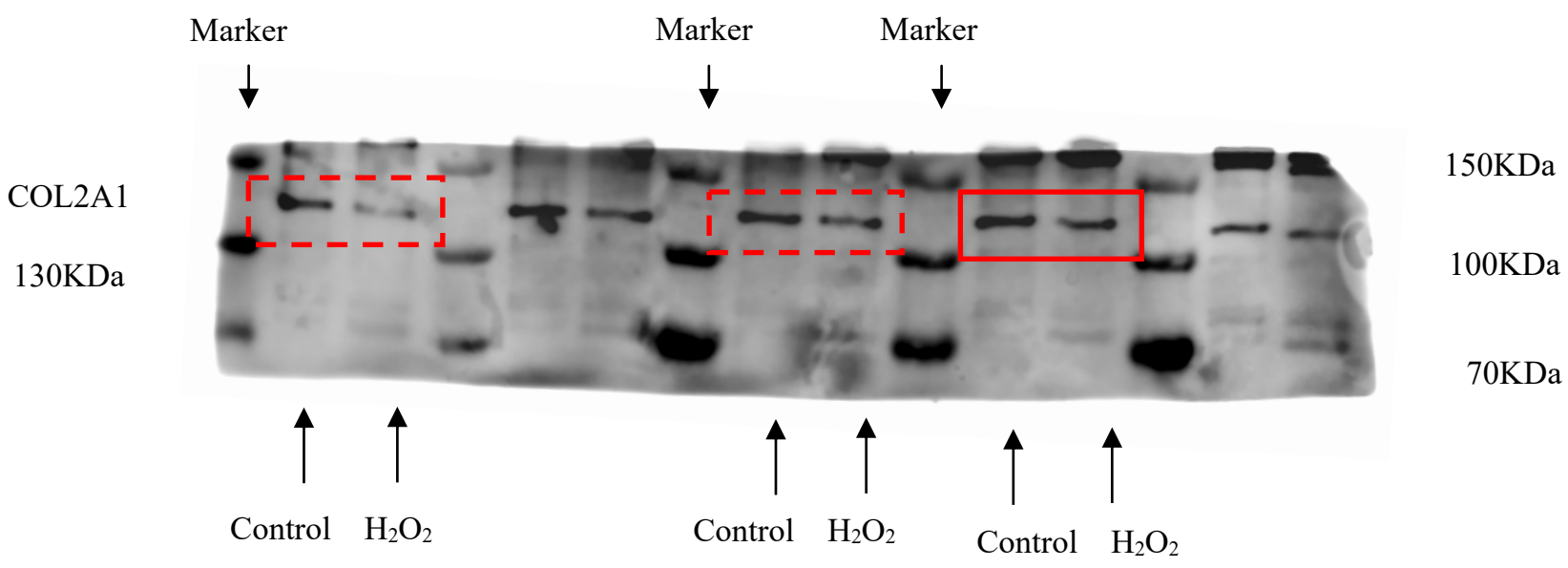
Marker
Marker
Marker
COL2A1
130KDa
150KDa
100KDa
70KDa
Control
H2O2
Control
H2O2
Control
H2O2

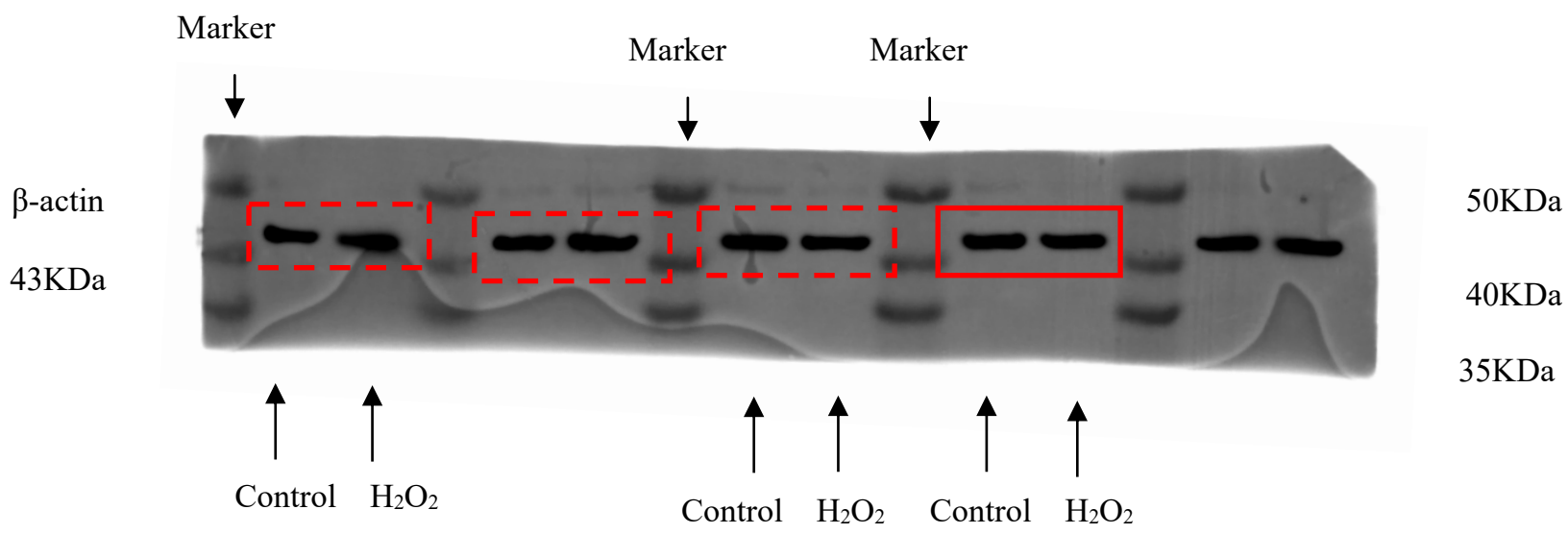
Marker
Marker
Marker
β-actin
43KDa
50KDa
40KDa
35KDa
Control
H2O2
Control
H2O2
Control
H2O2

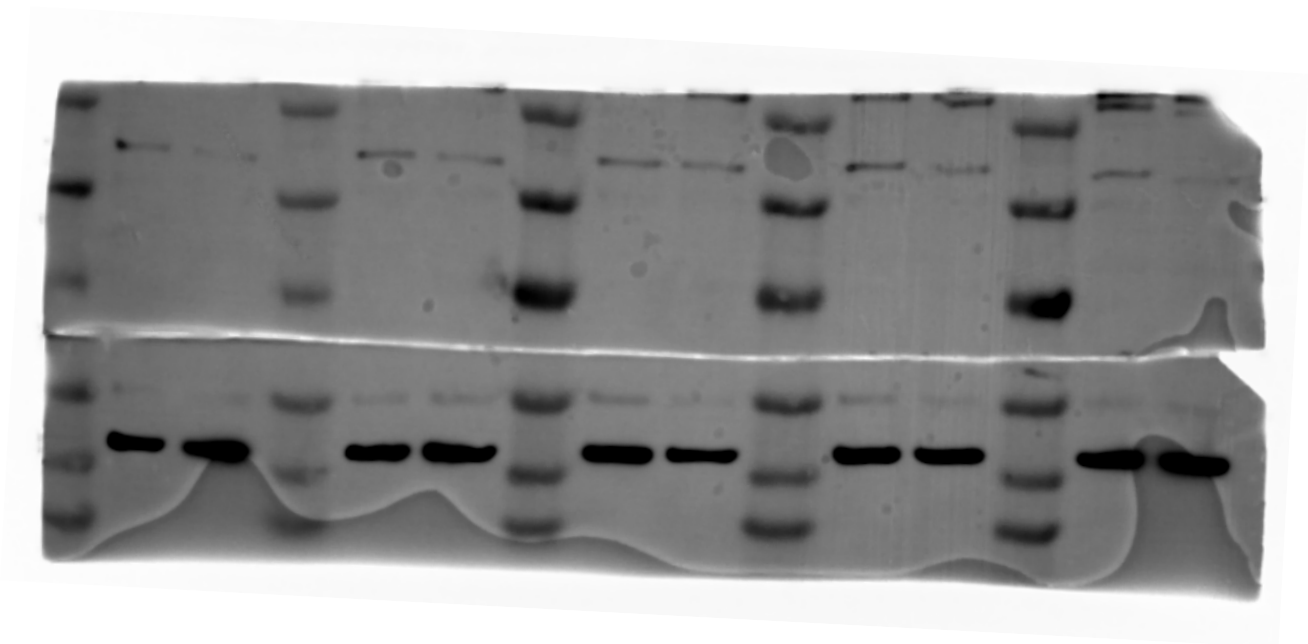

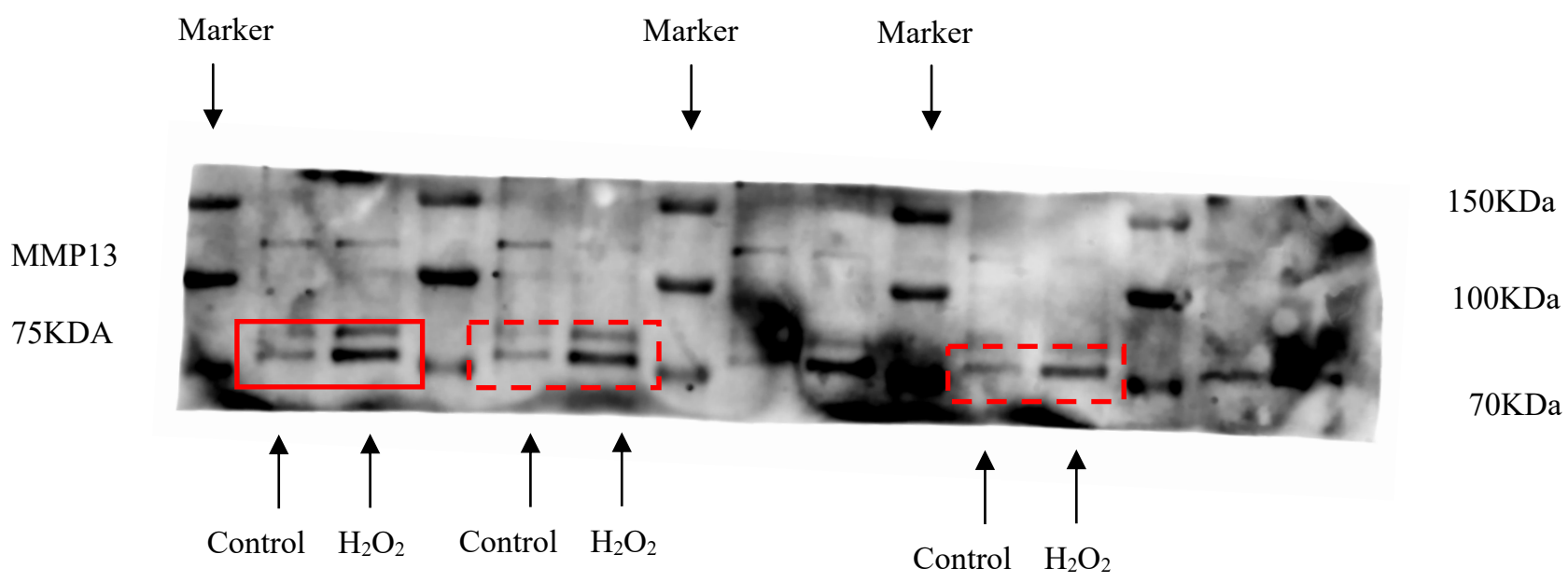
Marker
Marker
Marker
MMP13
75KDA
150KDa
100KDa
70KDa
Control
H2O2
Control
H2O2
Control
H2O2

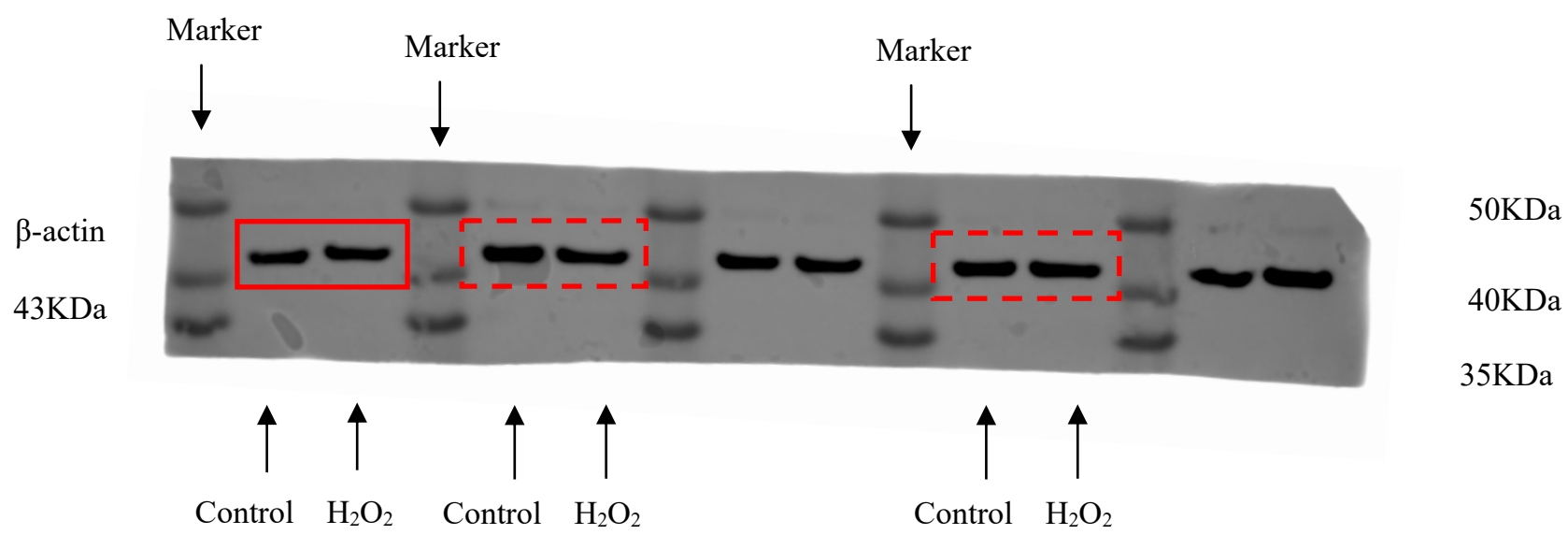
Marker
Marker
Marker
β-actin
43KDa
50KDa
40KDa
35KDa
Control
H2O2
Control
H2O2
Control
H2O2

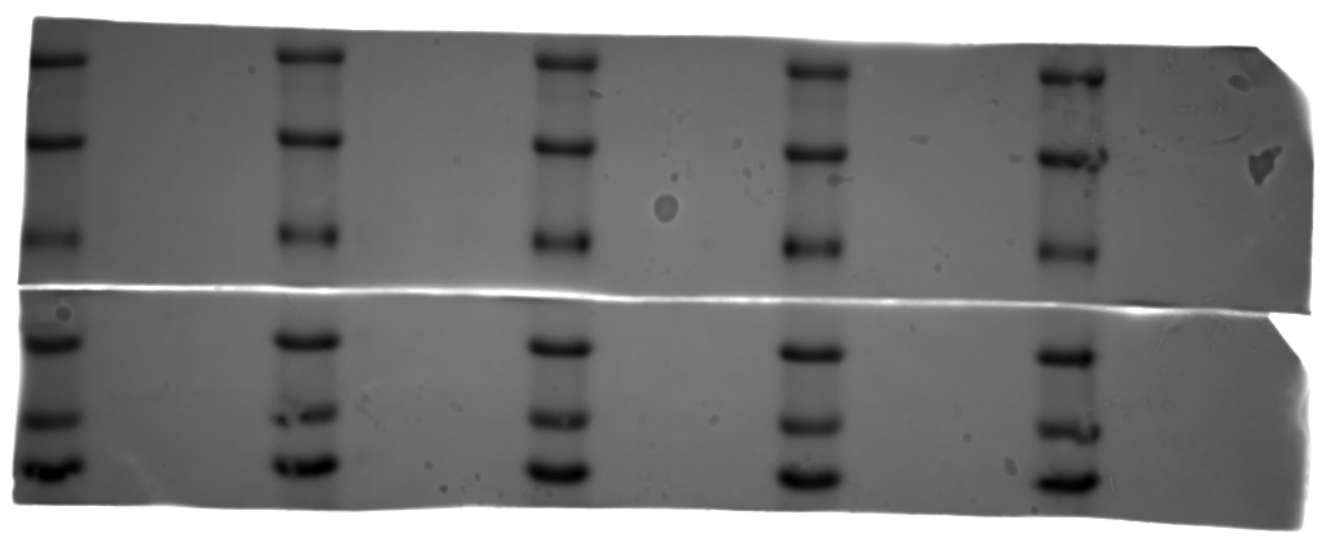

Supplement: Supplementary file 1 [file pone.0360537.s006.pdf]
